# Prognostic differential subpopulation classification and immunotherapy response prediction in pancreatic cancer patients based on the gene features of necrotizing apoptosis

**DOI:** 10.3389/fimmu.2025.1592231

**Published:** 2025-11-19

**Authors:** Kaili Liao, Zheng Fu, Xinrui Liu, Xiajing Yu, Linfeng Jin, Jinting Cheng, Dongyu Yang, Kun Ai, Ziqian Liu, Daixin Guo, Shuai Liu, Xiwen Yan, Zijia Li, Mingchen Xu, Xiya Yan, Jingyi Gan, Zhiwen Cheng, Wenqing Zhu, Mingxiu Cai, Wanqian Xu, Ziying Li, Jiasheng Xu, Xiaozhong Wang

**Affiliations:** 1 Jiangxi Province Key Laboratory of Immunology and Inflammation, Jiangxi Provincial Clinical Research Center for Laboratory Medicine, Department of Clinical Laboratory, The Second Affiliated Hospital, Jiangxi Medical College, Nanchang University, Nanchang, Jiangxi, China; 2 The First Clinical Medical College, Nanchang University, Jiangxi, Nanchang, China; 3 Queen Mary College, Jiangxi Medical College, Nanchang University, Nanchang, Jiangxi, China; 4 The Second Clinical Medical College, Nanchang University, Nanchang, Jiangxi, China; 5 School of Public Health, Nanchang University, Nanchang, Jiangxi, China; 6 The Forth Clinical Medical college, Nanchang University, Nanchang, Jiangxi, China; 7 Department of Colorectal Surgery, The Second Hospital of Zhejiang University School of Medicine, Key Laboratory of Cancer Prevention and Intervention, China National Ministry of Education, Hangzhou, Zhejiang, China; 8 Key Laboratory of Medical Molecular Biology, Zhejiang University, Hangzhou, Zhejiang, China

**Keywords:** carbohydrate sulfotransferase 11, multi-omics, necrotizing apoptosis, pancreatic cancer, spatial transcriptomics

## Abstract

**Introduction:**

This study aims to explore the prognostic significance of necroptosis-related genes in pancreatic cancer.

**Methods:**

First, clustering analysis was performed on 15 necroptosis-related genes, which led to the identification of two distinct NRG subtypes. Differential expression analysis revealed 495 genes associated with prognosis, which were subsequently used for a second round of clustering. Next, a prognostic model was constructed using seven key genes, and patients were classified into high-risk and low-risk groups. External cohort data were used to validate the prognostic model. Spearman correlation analysis was conducted to examine the relationship between the most important biomarker, CHST11, and the 15 NRG genes. Additionally, three single-cell datasets, along with Mendelian randomization and spatial transcriptomics analyses, were utilized to further investigate the associations between CHST11, immune therapy, immune cells, and malignant epithelial cells.

**Results:**

NRGcluster A and geneCluster B largely overlapped, with most patients classified into the low-risk group. Among the 15 NRG genes, 11 exhibited significant expression differences between the high-risk and low-risk groups. CHST11 was identified as the most important prognostic biomarker and showed significant correlations with 13 NRG genes. Further analysis revealed potential mechanisms of action for CHST11.

**Discussion:**

This study, through multi-omics data, reveals that CHST11 may be associated with necroptosis and is closely related to the malignant prognosis of pancreatic cancer.

## 1 Introduction

According to data from the Global Cancer Observatory (GLOBOCAN) 2022 (1), there were 510,992 new cases of pancreatic cancer and 467,409 deaths due to this disease worldwide in 2022. Pancreatic cancer is characterized by a very poor prognosis, with a persistently high mortality rate.

Immune checkpoint inhibitors (ICIs) have shown significant benefits in the treatment of certain cancer types, such as lung cancer and melanoma, but face numerous limitations in the treatment of pancreatic cancer (2). However, the discovery of necroptosis may open new avenues for the development of innovative treatment strategies (3). Cell death plays a crucial role in removing damaged cells to maintain physiological homeostasis, and it can also occur as an abnormal pathological response to damaging stimuli (4). When caspase-8 activity is lost, activation of tumor necrosis factor receptor (TNFR) family proteins (such as TNFR, FAS, TRAILR, and DR6) can trigger necroptosis, which subsequently activates receptor-interacting protein kinase 1-3 (RIPK1–RIPK3) (5–9). Among these, RIPK3 initiates the activation of mixed lineage kinase domain-like protein (MLKL). Once activated, MLKL aggregates and translocates to the plasma membrane, where it forms large pores, ultimately leading to necroptotic cell death. A clinical study of breast cancer tissue specimens showed that compared to adjacent non-cancerous tissues, the mRNA and protein expression levels of tumor necrosis factor alpha (TNFα), RIPK1, RIPK3, and MLKL were significantly elevated in breast cancer tissues.

Numerous studies have focused on the analysis of gene and non-coding RNA expression profiles to construct tumor classification systems and prognostic indicators to predict cancer patient survival outcomes and response to immunotherapy. In a recent study, researchers used necroptosis-related long non-coding RNA (lncRNA) to predict the prognosis of gastric cancer patients and classify molecular subtypes, successfully distinguishing "cold tumors" from "hot tumors" (10). Another team identified 12 necroptosis-related genes (NRGs) from a database and developed a prognostic evaluation tool for bladder urothelial carcinoma patients. These genes not only have important prognostic value but are also associated with immune subtypes and tumor stemness characteristics, providing valuable references for selecting optimal chemotherapy and immunotherapy regimens (11). Additionally, a recent study constructed a model based on necroptosis-related lncRNAs to predict and identify "cold tumors" and "hot tumors" in bladder urothelial carcinoma (12).

To date, numerous studies have focused on the role of necroptosis in pancreatic cancer; however, most current mechanistic research still centers around classical targets such as MLKL and RIPK3. Although some studies have explored necroptosis-related targets in pancreatic cancer using bioinformatics approaches from a clinical perspective, the mechanisms involved exhibit high complexity in clinical scenarios. Therefore, in-depth research into these mechanisms must be based on comprehensive exploration of relevant targets and extensive clinical correlation analysis. Current research has yet to provide sufficient candidate targets to fully explain this issue. This study aims to identify necroptosis-related targets and determine potential candidate targets closely associated with both necroptosis and pancreatic cancer prognosis, providing more directions and options for future research.

In our study, we first collected 67 necroptosis-related genes from previous literature and selected 15 common necroptosis genes. Based on these 15 genes, we performed consistency clustering analysis and divided the patients into two NRGclusters. We further identified differentially expressed genes, ultimately obtaining 495 genes associated with prognosis. Through a second consistency clustering analysis, we divided the patients into two geneClusters. Then, we selected 7 model genes from the 495 genes and constructed a prognostic model, dividing the patients into high-risk and low-risk groups based on the median risk score in the training set. We reviewed the correlation of features from the three rounds of grouping and found that NRGcluster A and geneCluster B exhibited a high degree of overlap and were almost entirely classified as the low-risk group. Additionally, among the high-risk and low-risk groups, 11 out of the 15 genes showed significant expression differences. We then used an external validation set to explore the prognostic characteristics of the 7 model genes, focusing on the most significant prognostic feature, CHST11. We first verified the significant positive correlation between CHST11 and the 15 genes (including GATA3 and FAS) through Spearman correlation analysis. We then analyzed immune treatment responses, the relationship between immune cells and non-immune cells, and the expression characteristics of CHST11 across three single-cell datasets. During this process, we also applied Mendelian randomization to screen for genes associated with pancreatic cancer from the differentially expressed genes and analyzed the prognostic characteristics of these genes and their association with CHST11 using transcriptome datasets. Finally, we visualized the spatial distribution characteristics of CHST11 and CTSC genes through spatial transcriptomics. In summary, this study used two rounds of consistency clustering analysis and a continuous biological screening system, ensuring that each extraction of prognostic features was associated with the 15 necroptosis-related genes, thus alleviating the feature dilution problem present in previous studies. Moreover, we revealed the prognostic role of CHST11 in pancreatic cancer through multi-omics analysis and explored its potential synergistic effects with immune regulation, cellular exhaustion, and CTSC.

## 2 Methods

### 2.1 Copy number variation of necroptosis genes

A total of 67 necroptosis genes were screened *via* GeneCards database (https://www.genecards.org) (**Supplementary Table 1**). Pancreatic adenocarcinoma (PAAD) CNV data were retrieved from TCGA, and gene-level annotation was conducted using CNTools (v1.64.0) (13) to obtain copy number values of 67 genes in 171 tumor samples. Genes were classified by log_2_ (copy number ratio): log_2_ ratio > 0.3 for copy number gain, and log_2_ ratio < -0.3 for copy number loss. The gain/loss frequencies of each gene across samples were calculated; ggplot2 (14) was used to visualize CNV frequencies (gain in red, loss in green) *via* scatter plots, showing population-level CNV distribution of different genes.

### 2.2 Dataset merging, batch effect removal and survival analysis of necroptosis genes

Raw count matrices of PAAD were downloaded from TCGA-PAAD and GSE62452 databases, with shared genes retained for merging. Batch effects were removed using the ComBat function (15), and the processed data were subjected to log_2_ transformation and normalization. Combining survival information and transcriptome data of 243 PAAD patients from TCGA-PAAD and GSE62452, 15 common genes were screened from 67 NRGs within the gene intersection of the two datasets. Univariate Cox regression analysis was then performed on these 15 genes.

### 2.3 Consensus clustering analysis of necroptosis genes

Consensus clustering of 15 NRGs was performed *via* the ConsensusClusterPlus package (16); patients were divided into two NRGClusters based on the result with the highest intra-group and lowest inter-group correlation. Prognostic differences between clusters were compared using the Kaplan-Meier (KM) method. Principal component analysis (PCA) was used to reduce the dimensionality of NRG expression matrices for verifying grouping reliability. DESeq2 (17) was applied for differential gene analysis (absolute FC > 1, *P* < 0.05), identifying 930 differential genes. GSVA (18) evaluated preset functional gene set enrichment, while single-sample GSVA (ssGSEA) (19) calculated infiltrating immune cell abundance and immune pathway activity. GO (20) and KEGG (21) analyses of the 930 differential genes were conducted using the clusterProfiler package.

### 2.4 Screening of prognostic genes and secondary clustering analysis

To further screen prognostic genes, 930 differentially expressed genes were first subjected to univariate COX regression analysis, with 495 candidate genes identified using *P* < 0.05 as the threshold. To verify the association between these 495 genes and 15 necroptosis genes, a second consensus clustering analysis was performed based on the expression levels of candidate genes. Patients were divided into two geneCluster groups according to the result with the highest intra-group correlation and most significant inter-group difference. Prognostic differences between the two groups were compared using the KM method. Subsequent differential expression analysis of 15 NRGs between geneCluster groups identified 13 genes with significant differential expression.

### 2.5 Screening of signature genes and construction of prognostic model for pancreatic cancer patients

Given the potential association between 495 candidate genes and 15 NRGs, least absolute shrinkage and selection operator (LASSO) regression was performed to screen 7 genes for constructing a prognostic signature, simplifying the model and extracting core features. Risk scores were calculated *via* multivariate Cox regression based on the expression levels of these 7 genes; patients were divided into high- and low-risk groups using the median risk score of the training cohort as the cutoff. The KM analysis was used to assess survival differences between the two groups, and receiver operating characteristic (ROC) curve with area under the curve (AUC) verified the predictive performance of the model. Additionally, a nomogram was constructed to predict survival probability by integrating patients’ tumor stage, grade, and risk score.

### 2.6 Association of prognostic model with two clustering results and necroptosis-related genes

To explore the association between prognostic risk scores, dual clustering results, and NRGs, the ggalluvial package (22) was used to generate a Sankey diagram, visualizing connections among NRG groups, geneCluster groups, risk stratification (high/low-risk groups), and survival status. Wilcoxon test was then applied to compare risk score differences between NRGCluster and geneCluster, as well as to assess expression differences of 15 NRGs between high- and low-risk groups.

### 2.7 External validation of survival analysis and pathway correlation analysis

To further validate the reliability of the survival analysis results, we independently validated the seven signature genes in an external validation set. The external validation data were sourced from the integration of GEO data by Máté Posta et al., including 12 GEO datasets such as GSE84219, GSE78229, and GSE179351, as well as four pancreatic cancer datasets from the International Cancer Genome Consortium (ICGC) data portal (23). In a total of 1237 clinical samples, we used the median expression value of each gene as the grouping threshold to perform single - gene Kaplan–Meier survival analysis respectively. Considering the potential bias brought by the integrated dataset itself, we selected the CHST11 gene with the most significant prognostic difference and divided the high- and low-expression groups using the median expression of CHST11 as the threshold in the pancreatic cancer datasets GSE85916, GSE28735, and GSE57495, performing Kaplan–Meier survival analysis. In addition, to further explore the prognostic value of the seven signature genes, we integrated the TCGA pancreatic cancer expression matrix and the GTEx pancreatic tissue expression matrix, and compared their expression differences in 179 pancreatic cancer samples and 171 normal tissues (24). Subsequently, we used Spearman correlation analysis to explore the association between the CHST11 gene with the most significant prognostic characteristics and the 15 NRGs.

### 2.8 Evaluation of tumor microenvironment in high- and low-risk groups

To explore the relationship between risk score and TME, the CIBERSORT (25) was first used for immune infiltration analysis of transcriptome data from high- and low-risk groups, quantifying the abundance distribution of 22 immune cell subsets; Spearman correlation analysis was applied to explore the association between 22 immune cells and risk score. Correlations between immune cell abundance and 7 signature genes were calculated to assess potential links between gene expression and immune microenvironment. The ESTIMATE (26) was used to obtain StromalScore, ImmuneScore, Tumor Purity, and EstimateScore for each sample. PCA was then applied for dimensionality reduction and visualization of TME features to compare distribution differences between high- and low-risk groups, while Wilcoxon rank-sum test was used to evaluate inter-group TME differences. The maftools package (27) was used to generate Mutation Annotation Format (MAF) files from mutation data, based on which Tumor Mutation Burden (TMB) scores were calculated. Tumor stem cell characteristics were characterized by downloading ssRNA from TCGA. Finally, Pearson correlation coefficients between risk score and TMB/ssRNA were calculated.

### 2.9 Drug sensitivity analysis

IC50 (half-maximal inhibitory concentration), an important pharmacological indicator for measuring drug activity, refers to the drug concentration required to inhibit 50% of cancer cell growth. The pRRophetic package (28) was used to predict and calculate the IC50 values of multiple chemotherapeutic drugs in high- and low-risk group samples, so as to evaluate the potential relationship between risk score and chemotherapeutic drug sensitivity.

### 2.10 Single-cell analysis of pancreatic cancer immunotherapy group

Single-cell analysis of the immunotherapy group was performed on the OmniBrowser platform (https://omnibrowser.abiosciences.cn). Relevant data of GSE150176 were retrieved on the platform, and the platform's differential expression analysis tool was used to compare the expression changes of signature genes in different cell subsets and before/after immunotherapy in pancreatic cancer tissues. All analysis steps were completed in the same platform environment.

### 2.11 Single-cell analysis of pancreatic cancer patients

The GSE155698 dataset was downloaded from the GEO database, containing single-cell transcriptome sequencing data from 16 pancreatic cancer tissue samples and 3 normal pancreatic tissue samples. All analyses were performed in R (v4.3.3). The Seurat (v4.3.0) package (29) was used to read and preprocess the sequencing matrix. Cells with fewer than 250 or more than 2,500 detected genes, fewer than 500 UMIs, or over 15% mitochondrial gene content were removed. In addition, cells with more than 3% ribosomal and less than 0.1% hemoglobin gene proportions were retained to eliminate blood contamination and ensure high-quality, transcriptionally active cells. Data were then normalized using the NormalizeData function. In feature selection, highly variable genes were identified using the vst method in FindVariableFeatures; data were scaled with ScaleData, regressing out cell cycle scores to eliminate cell cycle effects. PCA was performed *via* RunPCA, with principal components accounting for 90% cumulative variance explained selected for downstream analysis. The Harmony algorithm was used to integrate and correct batch effects between samples. After batch effect correction, FindNeighbors was used to construct a cell neighbor graph, and Louvain clustering was performed using FindClusters. Combined with marker genes, cells were annotated into 13 subpopulations. Based on CHST11 expression, naïve T cells and T cells were further divided into positive and negative cells, with expression differences of 15 NRGs compared between groups. Additionally, CellChat (v2.1.2) (30) was used with Seurat-processed expression matrices and cell annotations for cell-cell communication analysis; Monocle2 (31) combined with Seurat was used for pseudotime analysis to infer dynamic trajectories of cell state transitions.

### 2.12 Mendelian randomization

We used the FindMarkers function in the Seurat (v4.3.0) package to perform differential expression analysis using the Wilcoxon test, and set the threshold as: the absolute value of log2 fold change (FC) was greater than 0.25 and the p-adjust was less than 0.05. Based on this, the differentially expressed genes in positive/negative T cells and positive/negative naïve T cells were screened out respectively. Then, we selected the eQTL data (P < 5 × 10^-8^) that were significantly associated with gene expression within the ±1 Mb region upstream and downstream of the target gene. To ensure the independence of instrumental variables, we performed LD (linkage disequilibrium) pruning, setting r² < 0.01 and the window size as 1000 kb, and screened strong instrumental variables by calculating the F-statistic, retaining the instrumental variables with F > 10. Subsequently, we extracted the pancreatic cancer-related outcome datasets from the OPENGWAS database (https://opengwas.io/), including BBJ-140, IEU-822, GCST90018673, and GCST90018893. Finally, the TwoSampleMR (v0.6.12) (32) package was used to perform Mendelian Randomization (MR) analysis, and the validity of instrumental variables was evaluated through heterogeneity and pleiotropy tests. We screened genes with a P-value < 0.05 of the IVW method as the significance threshold. Subsequently, the Pearson correlation coefficients between these genes and the CHST11 gene in the TCGA dataset were calculated through the GEPIA2 database (33), and the prognostic values of the genes significantly associated with CHST11 were further explored in the GSE71729, TCGA-PAAD, and GSE62452 datasets.

### 2.13 Protein-protein interaction and RNA-binding protein analysis

To explore the interaction among candidate genes, the STRING database (34) was used to construct a PPI network. During the analysis, the minimum interaction confidence threshold was set to 0.4 (medium confidence), and isolated nodes were removed to obtain a biologically meaningful interaction network. The PPI network was then visualized using Cytoscape software (v3.9.1) (35) to further demonstrate the potential interaction relationships among these genes. Meanwhile, to explore the potential regulatory relationship between candidate genes and RBPs, interaction data between RBPs and target genes were retrieved from ENCORI (StarBase) data (36), and an RBP–mRNA interaction network was constructed using Cytoscape.

### 2.14 Single-cell analysis of non-immune cells and spatial transcriptomics in pancreatic cancer tissues

The single-cell dataset of non-immune cells in pancreatic cancer tissues (GSE194247) was preprocessed following the aforementioned pancreatic cancer immune cell single-cell analysis procedure. Marker genes from previous literature (37) were used for subpopulation annotation; the above-described method was applied to screen differential genes of malignant epithelial cells, followed by another Mendelian randomization analysis. For spatial transcriptomics (GSE235315), Seurat (v4.3.0) was used for data processing. After normalization *via* SCTransform, dimensionality reduction and clustering were performed with RunPCA, FindNeighbors, FindClusters, and RunUMAP; spatially variable genes were screened using FindSpatiallyVariableFeatures. In single-cell deconvolution, the GSE194247 single-cell reference dataset was loaded to extract cell type annotations. FindTransferAnchors established anchors between single-cell and spatial data, and TransferData mapped cell type labels to spatial transcriptomic data for predicting spatial cell type distribution.

### 2.15 Molecular docking

We downloaded the drug-gene association files from the Drug SIGnatures DataBase (38), and performed enrichment analysis based on a custom gene set using the enricher() function of the R package clusterProfiler (v4.12.0) (39). A double screening criterion was adopted, with the p-value (pvalueFilter=0.05) and the adjusted p-value (adjPvalFilter=0.05). Subsequently, we downloaded the three-dimensional structures of the corresponding drugs from the Pubchem database (40), predicted the protein structure of CHST11 in the ALPHAFOLD database (41), and finally performed online molecular docking using CB-DOCK2 (42).

### 2.16 qPCR and immunohistochemistry

We extracted RNA from normal pancreatic cells HPDE6-C7 and pancreatic cancer cells CFPAC-1, respectively. We took 1 µg of RNA and used a reverse transcription kit (Takara Biomedical Technology, China) for reverse transcription. After synthesizing cDNA, it was diluted for later use. For qPCR, the SYBR Green fluorescent dye system (Takara Biomedical Technology, China) was used. The 20 µL reaction system included 10 µL of SYBR Green Master Mix, 0.6 µL of forward primer (10 µM), 0.6 µL of reverse primer (10 µM), 2 µL of diluted cDNA template, and RNase-free water was added to make up to 20 µL. After the PCR amplification was completed, a melting curve analysis was performed to verify the specificity of the amplification products. The primer sequences are in the **Supplementary Table 2**. The immunohistochemistry (IHC) data were obtained from the Human Protein Atlas (HPA) database (https://www.proteinatlas.org).

## 3 Results

### 3.1 Prognostic correlation study of pancreatic cancer patients based on necroptosis genes

**Figure 1:**
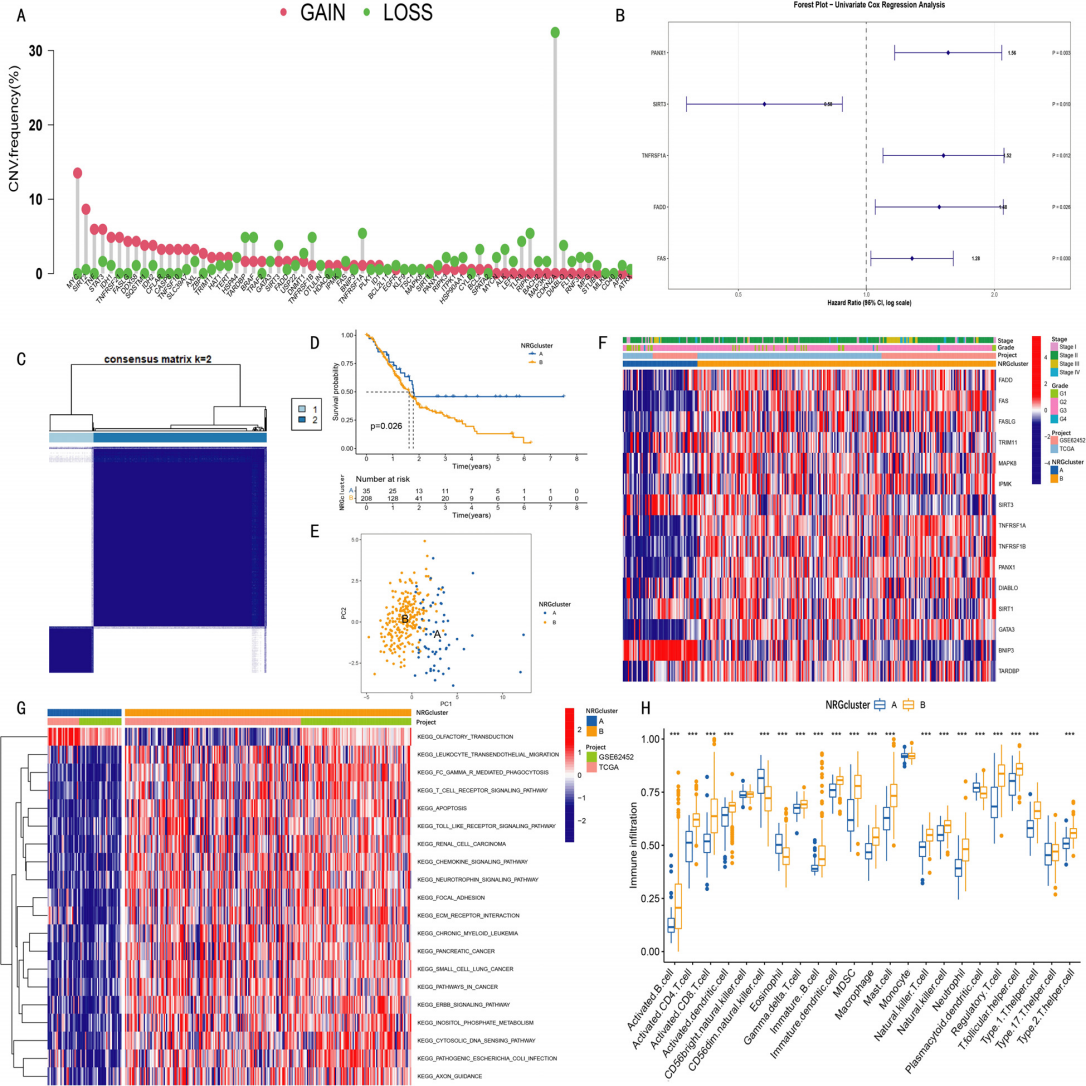
Clustering analysis of necroptosis-related genes (NRGs) in pancreatic cancer patients and the survival and clinical characteristics among different clusters. **(A)** Copy number variation (CNV) amplification and deletion frequencies of 67 necroptosis-related genes (NRGs) in TCGA pancreatic cancer cohort; **(B)** Among 15 NRGs used for clustering analysis, five showed statistical significance (p < 0.05) in univariate Cox analysis; **(C)** Consensus clustering identified two NRG clusters (NRGcluster A/B); **(D)** Kaplan-Meier curves showed that patients in NRGcluster A had longer survival time and higher survival probability than those in cluster B (p = 0.026); **(E)** Principal component analysis (PCA) revealed distinct separation between the two NRG clusters; **(F)** Heatmap displaying associations among NRG clusters, clinical features, and NRG expression levels in pancreatic cancer patients; **(G)** Gene set variation analysis (GSVA) revealed enriched pathways in different NRG clusters; **(H)** Single-sample gene set enrichment analysis (ssGSEA) indicated differences in immune cell infiltration between NRG clusters (***p < 0.001).

The copy number variation (CNV) data of pancreatic cancer patients were obtained from The Cancer Genome Atlas (TCGA) database. In this study, the correlation between 67 necroptosis-related genes (NRGs) and patient prognosis was first analyzed. The results of CNV frequency analysis (**Figure 1A**) showed that among the 67 genes, the amplification of MYC gene was the most common, followed by SIRT2, STST3 and TNF genes, while the copy number deletion of CDKN2A, RIPK1 and TNFRSF1A genes was widespread. Subsequently, the GSE62452 dataset (including 69 tumor samples and 61 normal control samples) was merged with TCGA data in this study, and 15 NRGs shared by the two datasets were screened for subsequent analysis. Univariate Cox analysis was performed on 243 pancreatic cancer patients with complete survival information. The results showed that among the 15 NRGs, 5 were significantly correlated with patient prognosis (**Figure 1B**).

### 3.2 Consensus clustering analysis based on 15 NRGs

Clustering analysis was performed using the expression data of 15 necroptosis-related genes (NRGs). The optimal number of clusters (k value) was determined by progressively increasing k, with the highest intra-cluster correlation and lowest inter-cluster correlation observed at k=2 (**Supplementary Figure S1**). Accordingly, pancreatic cancer patients were divided into two groups: NRGCluster A and NRGCluster B (**Figure 1C**).

Kaplan-Meier survival analysis demonstrated that patients in NRG Cluster A had longer overall survival and higher survival probability than those in Cluster B (p=0.026) (**Figure 1D**). Principal component analysis (PCA) further (**Figure 1E**) confirmed a clear separation between the two NRG clusters in the 2D coordinate space. The heatmap (**Figure 1F**) illustrated the relationships among NRG clusters, clinical characteristics of pancreatic cancer patients, and the expression pattern of 15 NRGs, indicating significant differences between Cluster A and Cluster B.

Gene Set Variation Analysis (GSVA) results (**Figure 1G**) demonstrated that NRG Cluster B was significantly enriched in immune regulation and inflammation-related signaling pathways, including leukocyte transendothelial migration, Fcγ receptor-mediated phagocytosis, T cell receptor signaling, Toll-like receptor signaling, and chemokine signaling pathways. Further validation using Single Sample Gene Set Enrichment Analysis (ssGSEA) (**Figure 1H**) revealed that Cluster B exhibited a generally higher degree of immune cell infiltration, particularly in activated B cells, activated CD4^+^T cells, activated CD8^+^ cells, activated dendritic cells, macrophages, natural killer (NK) cells, and regulatory T cells. However, the infiltration levels of some immune cells (CD56dim NK cells, eosinophils, and plasmacytoid dendritic cells) were lower in Cluster B than in Cluster A.

### 3.3 Consensus clustering analysis of prognosis-related differentially expressed genes based on NRG clusters

**Figure 2:**
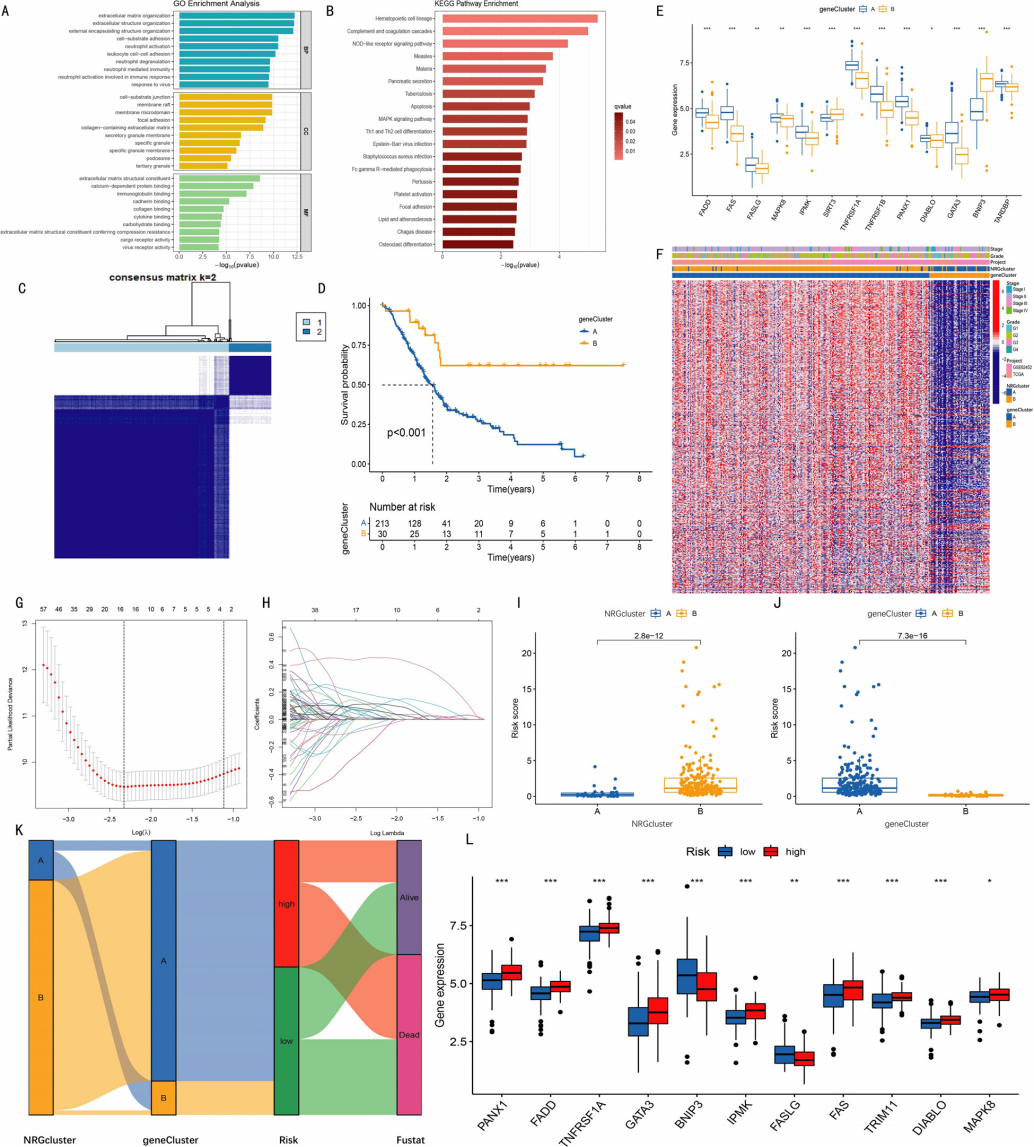
Clustering analysis of geneClusters and construction of the prognostic model. **(A)** Gene Ontology (GO) enrichment analysis revealed biological processes (BP), cellular components (CC), and molecular functions (MF) associated with differentially expressed genes (DEGs) between two NRGclusters; **(B)** Kyoto Encyclopedia of Genes and Genomes (KEGG) pathway enrichment analysis identified pathways enriched by DEGs; **(C)** Based on expression levels of 495 PRDEGs (prognosis-related DEGs identified by univariate Cox regression), two geneClusters were established; **(D)** Kaplan-Meier curves indicated higher survival rates in cluster B compared with cluster A (p < 0.001); **(E)** Expression levels of 13 out of 15 NRGs differed significantly between the two geneClusters; **(F)** Heatmap showing relationships among NRGcluster, geneCluster, risk, and s expression; **(G)** K-fold cross-validation for LASSO parameter optimization (x-axis: log λ; y-axis: partial likelihood deviance); **(H)** LASSO coefficient profiles (x-axis: log λ; y-axis: gene coefficients); **(I–J)** Differences in risk scores between two NRGclusters **(I)** and two geneClusters **(J)**; **(K)** Sankey diagram illustrating relationships among NRGcluster, geneCluster, risk score, and survival status; **(L)** Among 15 NRGs, 11 showed significantly different expression levels between high- and low-risk groups (*p < 0.05, **p < 0.01, ***p < 0.001).

A total of 930 differentially expressed genes (DEGs) were identified between the two NRG clusters (**Supplementary Table 3**). Gene Ontology (GO) enrichment analysis of these 930 DEGs (**Figure 2A**) showed that the DEGs were involved in biological processes (BPs) such as extracellular matrix organization, extracellular structure organization, and external encapsulating structure organization. In terms of molecular functions (MFs), the DEGs were significantly associated with extracellular matrix structural constituent, calcium-dependent protein binding, and immunoglobulin binding. Kyoto Encyclopedia of Genes and Genomes (KEGG) pathway enrichment analysis further revealed that these DEGs were enriched in the hematopoietic cell lineage, complement and coagulation cascades, and NOD-like receptor signaling pathways (**Figure 2B**). Subsequently, 495 prognostic DEGs (PRDEGs) associated with pancreatic cancer prognosis were identified *via* univariate Cox regression analysis (**Supplementary Table 4**).

Based on the expression data of 495 PRDEGs, k=2 was selected as the optimal clustering variable (**Supplementary Figure S2**), and pancreatic cancer patients were divided into two groups: geneCluster A and geneCluster B (**Figure 2C**). Kaplan-Meier curves showed that the survival rate of patients in geneCluster B was significantly higher than that in geneCluster A (**Figure 2D**). Examination of NRG expression levels in the two gene clusters revealed that among the 15 necroptosis-related genes (NRGs), the expression levels of FADD, FAS, FASLG, MAPK8, TNFRSF1A, TNFRSF1B, PANX1, DIABLO, GATA3, IPMK, and TARDBP were increased in geneCluster B, while the expression levels of SIRT3 and BNIP3 were decreased (**Figure 2E**). A comprehensive heatmap illustrated the associations among the gene clusters, patients' clinical characteristics, PRDEG expression levels, and NRG clusters, highlighting distinct transcriptional and clinical profiles between geneCluster A and geneCluster B. (**Figure 2F**).

### 3.4 Construction and validation of risk model based on prognostic differentially expressed genes

To construct a prognostic model, LASSO regression was applied to the 495 PRDEGs (**Figures 2G-H**), ultimately identifying seven key genes (CHST11, SLC16A1, RHOF, ANO6, PAH, MAN1C1, SPRR1B) for inclusion in the risk scoring model. Subsequently, risk score differences between NRGcluster and geneCluster groups were compared. Boxplots showed that in NRGcluster, the risk score of cluster A was lower than that of cluster B; in geneCluster, the risk score of geneCluster A was higher than that of geneCluster B, with all differences reaching statistically significant (**Figures 2I-J**). A Sankey diagram was used to show the relationships among NRGcluster, geneCluster, high/low-risk groups, and survival status. The distributions of patients in NRGcluster A and geneCluster B were nearly identical, both corresponding to the low-risk group, suggesting a potential association between the seven model genes and the 15 necroptosis-related genes (**Figure 2K**). Further analysis of NRG expression between high- and low-risk groups revealed significant differences in 11 of the 15 genes. Specifically, PANX1, FADD, TNFRSF1A, GATA3, IPMK, and FAS were highly expressed in the high-risk group, while BNIP3 and FASLG were highly expressed in the low-risk group (**Figure 2L**).

### 3.5 Model performance validation

**Figure 3:**
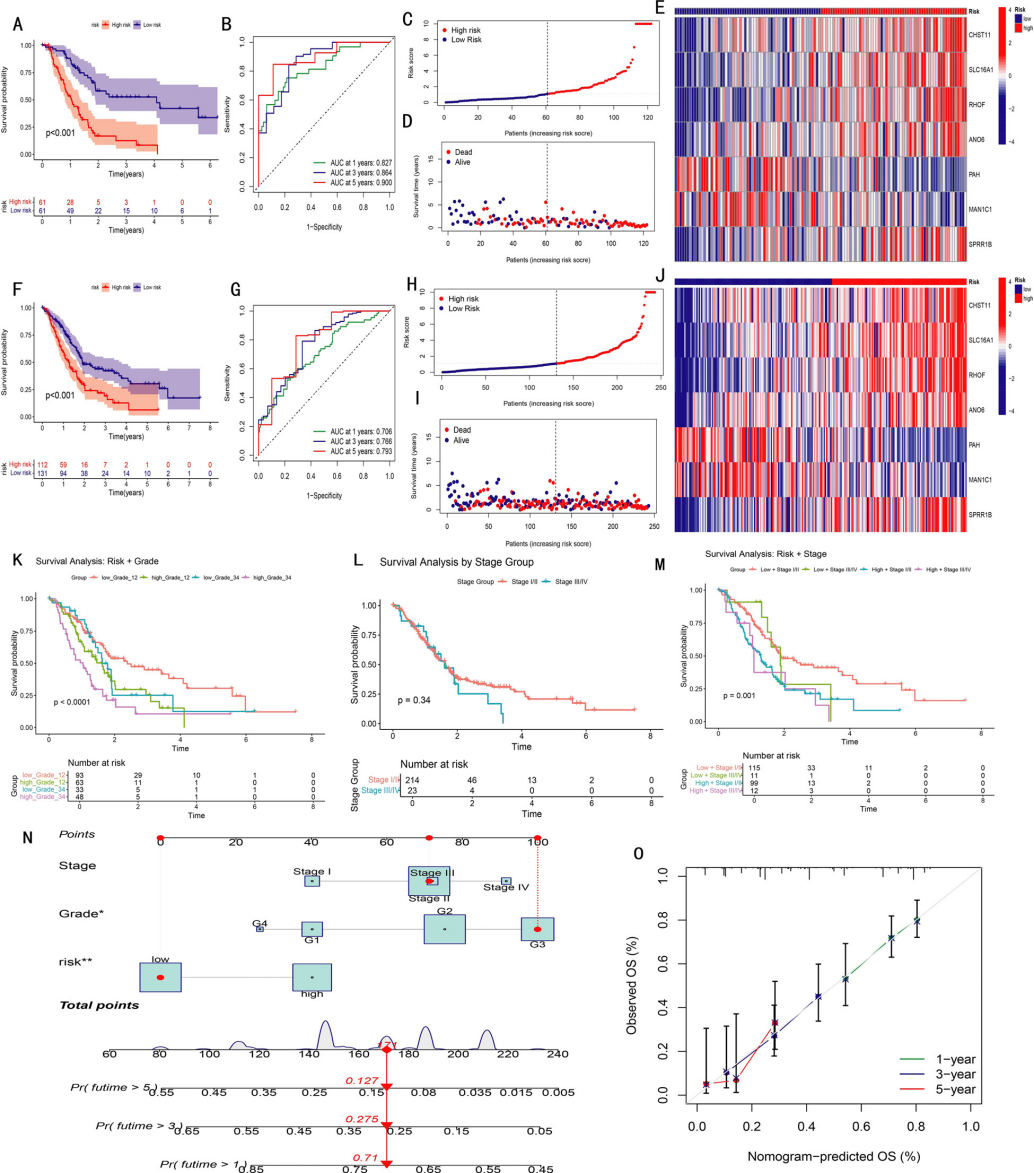
Validation and performance evaluation of the prognostic model in pancreatic cancer (PAAD). **(A, F)** Kaplan-Meier survival curves comparing high- and low-risk groups in the training set **(A)** and entire cohort **(F)**; **(B, G)** Time-dependent ROC curves for predicting 1-, 3-, and 5-year overall survival in training **(B)** and full **(G)** datasets (AUC values shown); **(C, H)** Distribution of risk scores in ascending order for patients in training **(C)** and full **(H)** datasets; **(D, I)** Distribution of survival status according to risk score in training **(D)** and full **(I)** datasets; **(E, J)** Heatmaps of the seven model genes in high- and low-risk groups, training **(E)** and full **(J)**; **(K–M)** Kaplan-Meier analyses stratified by Grade 1–2 vs. Grade 3–4 **(K)**, Stage I/II vs. Stage III/IV **(L)**, and combined stage and risk groups **(M)**; **(N)** Nomogram integrating risk score and clinical variables to predict overall survival; **(O)** Calibration curves showing agreement between predicted and observed survival probabilities.

Patients were ranked by risk scores (**Figures 3C, H**) and divided into high- and low-risk groups using the median score of the training set. Kaplan–Meier analysis revealed significantly poorer overall survival in the high-risk group across both training and combined cohorts (P < 0.001, **Figures 3A, F**). As risk scores increased, mortality also rose, with survivors clustering predominantly in the low-risk group (**Figures 3D, I**). Heatmaps revealed consistent trends: CHST11, SLC16A1, RHOF, ANO6, and SPRR1B were upregulated in the high-risk group, while PAH and MAN1C1 showed the opposite pattern (**Figures 3E, J**). ROC curves demonstrated strong predictive ability, with AUCs of 0.827, 0.864, and 0.900 for 1-, 3-, and 5-year survival in the training cohort, respectively, and similar results in the overall dataset (**Figures 3B, G**).

To assess model applicability across clinical subgroups, Kaplan–Meier analyses were performed in grade I–II vs. III–IV and stage I–II vs. III–IV subsets. The model effectively distinguished prognoses in all subgroups. Notably, staging alone failed to show significant survival differences (P = 0.34), but when combined with the risk score, four distinct prognostic groups emerged (P = 0.001), highlighting the model’s added prognostic value (**Figures 3K–M**).

A nomogram integrating stage, grade, and risk score was developed to predict individual survival probabilities, where total points estimated survival at specific time points (**Figure 3N**). Calibration curves demonstrated excellent concordance between predicted and observed outcomes (**Figure 3O**).

**Figure 4:**
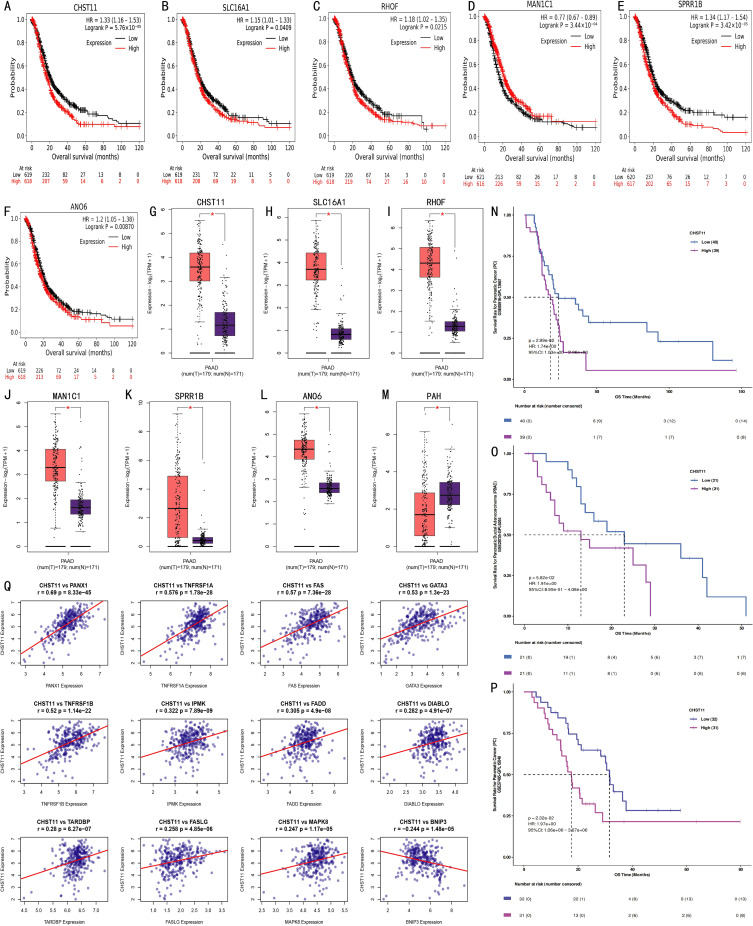
External validation of prognostic model genes. **(A–F)** Kaplan-Meier curves from Kaplan-Meier Plotter database showing associations between expression levels of CHST11, SLC16A1, RHOF, ANO6, MAN1C1, and SPRR1B (split by median expression) and survival outcomes of 1,237 pancreatic cancer patients; **(G–M)** Scatter plots comparing expression of CHST11, SLC16A1, RHOF, MAN1C1, SPRR1B, ANO6, and PAH in normal pancreatic tissues (GTEx, n = 171) and tumor tissues (TCGA, n = 179) (*p < 0.01); **(N–P)** Kaplan-Meier curves from GSE85916, GSE28735, and GSE57495 datasets showing survival differences between high and low CHST11 expression groups; **(Q)** Spearman correlations identifying 12 NRGs associated with *CHST11*.

To evaluate the independent prognostic value of the seven feature genes, survival analysis was performed for 1,237 pancreatic cancer cases using the Kaplan–Meier Plotter database. Based on median expression levels, CHST11, SLC16A1, RHOF, ANO6, MAN1C1, and SPRR1B were significantly associated with overall survival. Among these, MAN1C1 predicted a favorable prognosis, whereas the others correlated with poor outcomes (**Figures 4A–F**), consistent with model-based trends. Integrated analysis of GTEx (normal, n = 171) and TCGA (tumor, n = 179) datasets revealed higher expression of CHST11, SLC16A1, RHOF, MAN1C1, SPRR1B, and ANO6 in tumors (**Figures 4G–L**). In contrast, PAH expression was significantly lower in tumor tissues compared with normal counterparts (**Figure 4M**). Across the GSE85916, GSE28735, and GSE57495 datasets, high CHST11 expression consistently correlated with worse survival (**Figures 4N–P**). Spearman correlation further indicated that CHST11 expression was positively associated with multiple NRGs, suggesting a potential mechanistic link between necroptosis and pancreatic cancer progression (**Figure 4Q**).

### 3.6 Analysis of tumor microenvironment and drug sensitivity in high- and low-risk groups

**Figure 5:**
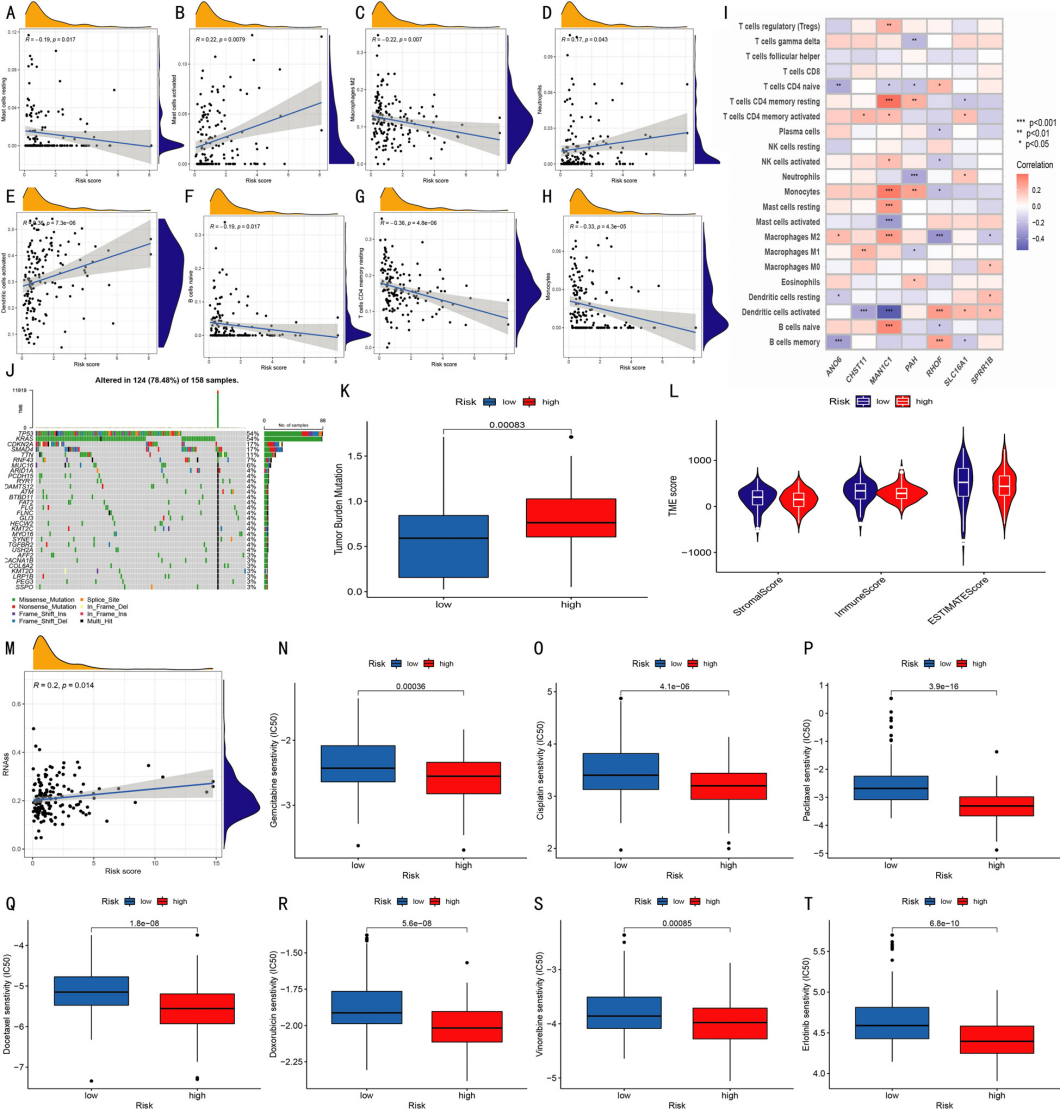
Prognostic characteristics of the model. **(A–H)** Correlations between infiltration levels of various immune cells and risk scores; **(I)** Correlations between 22 immune cell types and seven model genes (*p < 0.05, **p < 0.01, ***p < 0.001); **(J)** Waterfall plot of somatic mutations in 158 patients; **(K)** Tumor mutation burden (TMB) comparison between risk groups; **(L)** Violin plots show StromalScore, ImmuneScore, and ESTIMATEScore between groups; **(M)** Correlation analysis between RNA stemness score and risk score; **(N–T)** Boxplots show IC50 differences for Gemcitabine, Cisplatin, Paclitaxel, Docetaxel, Doxorubicin, Vinorelbine, and Erlotinib between high- and low-risk groups.

The correlation between immune cell infiltration and the risk score was analyzed (**Figures 5A–H**). Results showed that the abundances of naive B cells, M2 macrophages, resting mast cells, monocytes, and resting CD4 memory T cells were negatively correlated with the risk score, while activated dendritic cells, activated mast cells, and neutrophils were positively correlated. Further analysis revealed the correlation between 22 immune cells and 7 risk model genes (**Figure 5I**). In somatic mutation analysis of 158 pancreatic cancer patients (**Figure 5J**), the tumor mutation burden (TMB) in the high-risk group was significantly higher than that in the low-risk group (**Figure 5K**). Comparison of tumor microenvironment (TME) scores between the two groups showed no significant differences in StromalScore, ImmuneScore, or ESTIMATEScore (**Figure 5L**), nor in tumor purity (**Supplementary Figures S3**, **S4**). Additionally, RNA Stemness Score was significantly positively correlated with risk score (**Figure 5M**). Drug sensitivity analysis based on the pRRophetic algorithm showed significant differences in 70 drugs between the two groups (P < 0.05). Among them, Gemcitabine, Cisplatin, Paclitaxel, Docetaxel, Doxorubicin, Vinorelbine, and Erlotinib had significantly lower IC50 values in the high-risk group (**Figures 5N–T**), suggesting these drugs may be more suitable for high-risk patients. Other differential drugs are shown in **Supplementary Figure S5**.

### 3.7 Single-cell transcriptome analysis of mouse immunotherapy group

**Figure 6:**
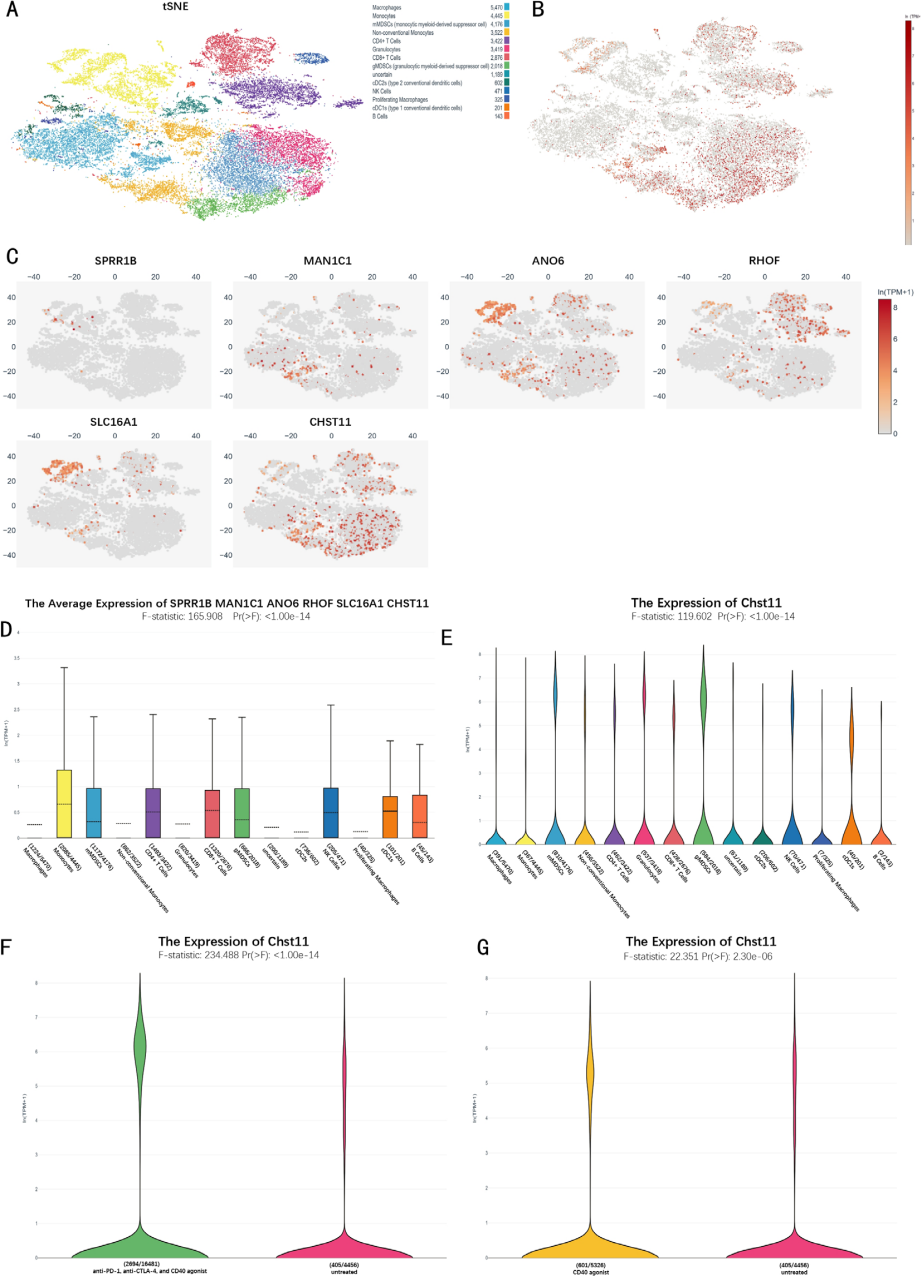
Single-cell transcriptomic analysis of the immunotherapy cohort in pancreatic cancer.**(A)** t-SNE plot of 14 cellular subclusters; **(B)** Overall expression of six model genes across all cells; **(C)** Expression of six model genes in specific cell subclusters; **(D)** Boxplot showing overall expression levels of the six model genes in immune cells; **(E)** Violin plot of CHST11 expression across cell types; **(F)** CHST11 expression significantly upregulated in CD40 agonist-treated group versus untreated (p < 0.001); **(G)** CHST11 expression significantly elevated in CD40 agonist, anti–PD-1+, and anti–CTLA-4 combination groups versus untreated (p < 0.001).

Single-cell RNA sequencing (scRNA-seq) analysis of the mouse immunotherapy cohort identified 14 cell subsets, including macrophages, monocytes, and monocytic myeloid-derived suppressor cells (mMDSCs). t-SNE plots visualized the expression distribution of 6 model genes across cell subsets (**Figures 6A–C**). Box plots further showed these genes' expression characteristics (**Figure 6D**). Violin plots revealed high CHST11 expression in T cells, NK cells, MDSCs, and cDC1s (**Figure 6E**). Additionally, CHST11 expression was significantly higher in the CD40 agonist group and combined therapy group than in the untreated group (**Figures 6F–G**).

### 3.8 Single-cell analysis of pancreatic cancer patients

**Figure 7:**
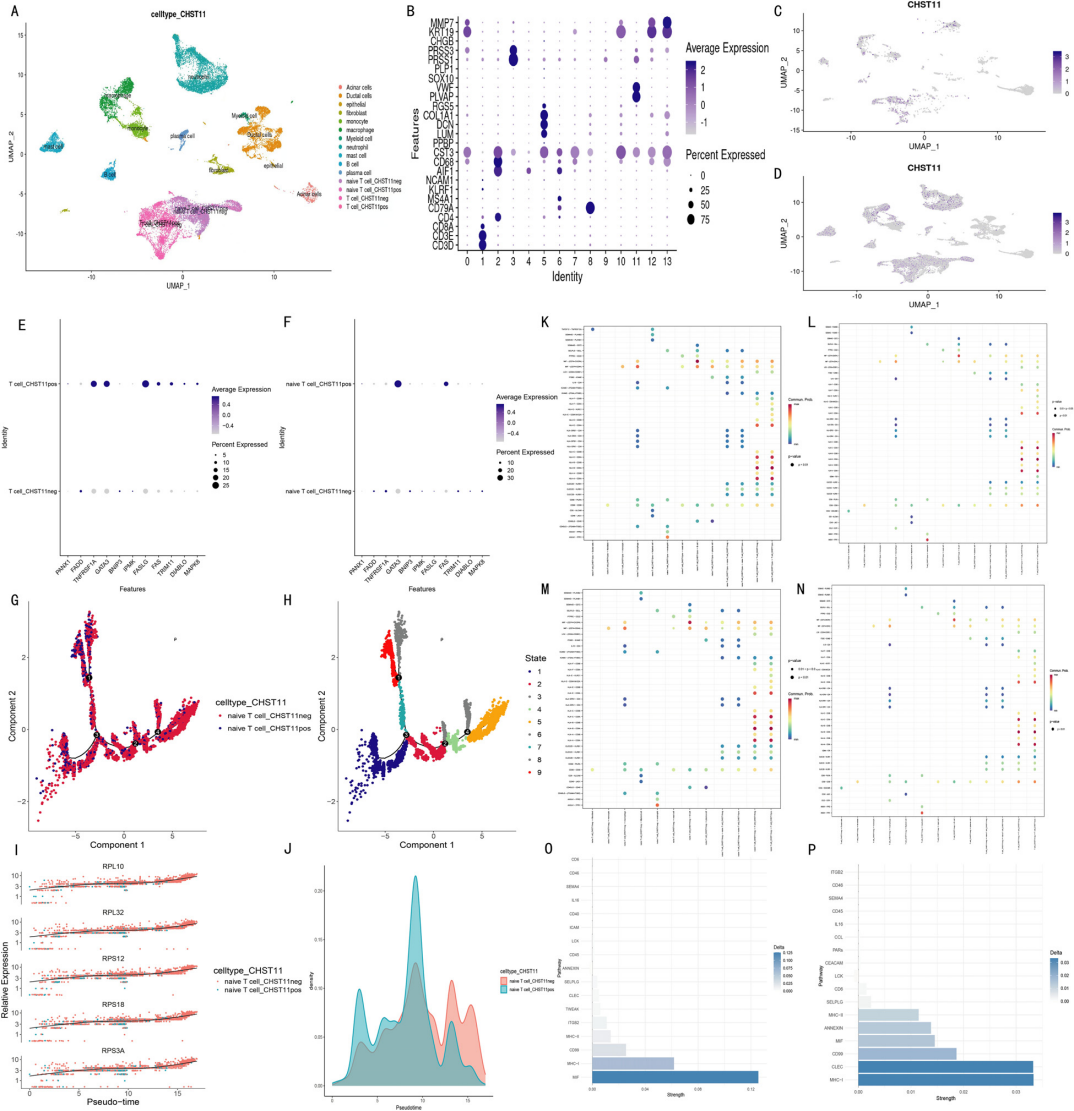
Single-cell transcriptomic analysis of pancreatic cancer patients. **(A)** UMAP plots identifying 15 cell subtypes based on marker genes; **(B)** Average expression and proportion of marker genes within each subtype; **(C)** UMAP of CHST11 expression in adjacent normal tissue; **(D)** UMAP showing CHST11 expression mainly in T cells, macrophages, and neutrophils in tumor tissue; **(E, F)** Expression of 15 NRGs in CHST11^+^ and CHST11^-^ T cells and naïve T cells; **(G, H)** Pseudotime analysis showing state distribution of naïve T cells with different CHST11 expression levels; **(I)** Dynamic expression changes of five core genes along pseudotime; **(J)** Cell density distribution of naïve T cells along pseudotime; **(K–N)** Bubble plots of ligand–receptor communication in *CHST11*^+^/^-^ naïve T and T cells; **(O)** Differences in communication strength within MIF, MHC-I, and CD99 pathways between CHST11^+^ and CHST11^-^ naïve T-cell subsets;**(P)** Differences in communication strength within CD99, MHC-I, and CLEC pathways between CHST11^+^ and CHST11^-^ T-cell subsets.

Single-cell data from tumor tissues of 17 pancreatic cancer patients and adjacent normal pancreatic tissues of 3 patients were preprocessed, and the expression distribution of 7 model genes across different cell subsets was visualized (**Supplementary Figure S6**). Combined with marker genes, cells were divided into 15 subsets (**Figures 7A, B**). In pancreatic cancer tissues, CHST11 expression was predominantly observed in T cells, macrophages, and neutrophils (**Figure 7D**), while in adjacent normal tissues, it was mainly expressed in T cells (**Figure 7C**).

Subsequently, we focused on characteristic analysis of T cells and naive T cells. Based on CHST11 expression, T cells were divided into CHST11^+^ T cells and CHST11^-^ T cells, and naive T cells into CHST11^+^ naive T cells and CHST11^-^ naive T cells. In CHST11^+^ T cells, the expression proportion and average expression level of GATA3, TNFRSF1A, and FASLG among 15 NRGs were significantly upregulated; the average expression level of FAS, TRIM11, DIABLO, and MAPK8 also increased (**Figure 7E**). In CHST11^+^ naive T cells, the expression proportion and average expression level of GATA3 were significantly elevated (**Figure 7F**). Pseudotime analysis showed that CHST11^+^ naive T cells were mainly distributed in State 1–4 (**Figures 7G, H**) and exhibited higher cell density in the early stage of Pseudotime (**Figure 7J**). **Figure 7I** shows the dynamic changes of five core genes in Pseudotime. Additionally, we compared cell communication patterns between CHST11^+^ and CHST11^-^ naive T cells, as well as between CHST11^+^ and CHST11^-^ T cells (**Figures 7K–N**). Results showed that CHST11^+^ naive T cells had significantly higher communication intensity in MIF and MHC-I pathways than the negative group (**Figure 7O**); CHST11^+^ T cells also had significantly upregulated communication intensity in MHC-I, CLEC, CD99, MIF, ANNEXIN, and MHC-II pathways (**Figure 7P**).

### 3.9 Mendelian randomization

Differentially expressed genes (DEGs) were identified between CHST11-positive and -negative subsets of T cells and naïve T cells. Expression quantitative trait loci (eQTLs) significantly associated with these differential genes were used as exposure variables, and BBJ-140, IEU-822, GCST90018673, and GCST90018893 as outcomes for Mendelian randomization analyses. Ten pancreatic cancer-related genes were screened using the inverse variance weighted (IVW) method with *P* < 0.05 (**Supplementary Table 5**).

**Figure 8:**
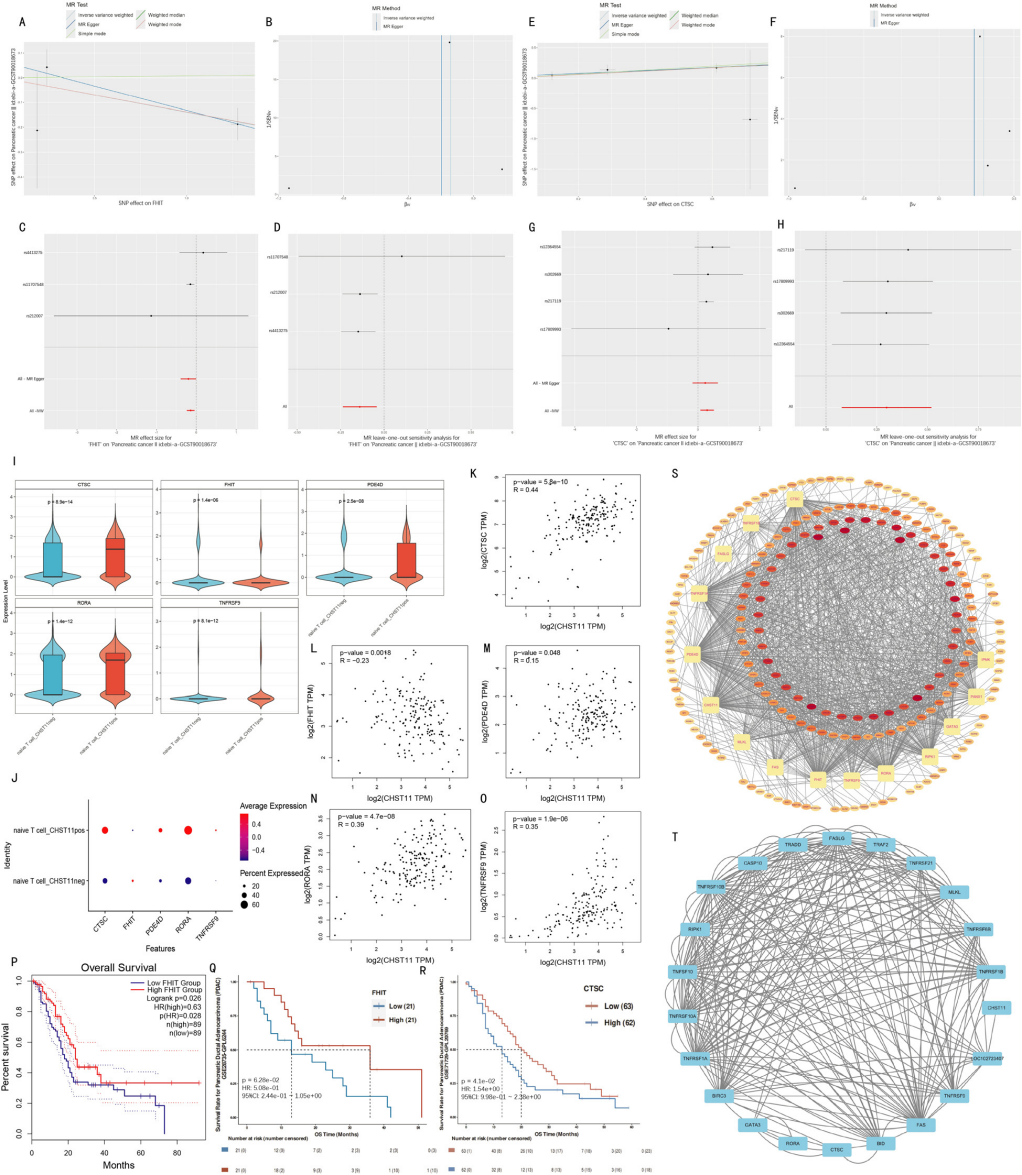
Mendelian randomization analysis and validation. **(A–D)** Mendelian randomization results for FHIT (exposure) and ebi-a-GCST90018673 (outcome); **(E–H)** MR results for *CTSC* (exposure) with ebi-a-GCST90018673 (outcome); **(I–J)** Violin and bubble plots showing differential expression of significant MR genes between CHST11^+^/^-^ naïve T cells; **(K–O)** Pearson correlations between CHST11 and five genes in TCGA-PAAD dataset; **(P–R)** Survival analyses stratified by FHIT and CTSC median expression levels; **(S)** RBP interaction network of 15 genes; **(T)** Protein–protein interaction (PPI) analysis with confidence score > 0.4.

Subsequently, Pearson correlation analysis was performed in pancreatic adenocarcinoma (PAAD) patients from The Cancer Genome Atlas (TCGA) to explore correlations between these 10 genes and CHST11. Five genes (CTSC, FHIT, PDE4D, RORA, and TNFRSF9) were selected with *P* < 0.05. **Figures 8A–H** visualized Mendelian randomization results with FHIT/CTSC as exposures and the pancreatic cancer GWAS dataset ebi-a-GCST90018673 as the outcome.

FHIT was identified as a protective factor for pancreatic cancer *via* Mendelian randomization, showing a significant negative correlation with CHST11 in TCGA (*R* = -0.23) and lower expression in naïve T cell_ CHST11pos than in naïve T cell_ CHST11neg (**Figures 8I–J, L**). Survival analysis revealed better survival in patients with high FHIT expression (**Figures 8P–Q**).

In contrast, CTSC was a risk factor for pancreatic cancer *via* Mendelian randomization, with a significant positive correlation with CHST11 in TCGA (*R* = 0.44) and higher expression in naïve T cell_pos than in naïve T cell_neg (**Figures 8I–K**). Patients with low CTSC expression had better survival (**Figure 8R**).

RNA-binding protein (RBP) interaction analysis was conducted for 15 genes including CTSC, FHIT, MLKL, FAS, and CHST11. Forty-two RBPs potentially bound to over 10 genes; among these, 5 RBPs (DDX3X, ELAVL1, HNRNPA2B1, IGF2BP3, and TARDBP) showed significant correlations with 14 genes, suggesting their key roles in post-transcriptional regulation of candidate genes (**Figure 8S**; detailed interactions in **Supplementary Table 6**).

Protein–Protein Interaction (PPI) analysis was performed with a confidence threshold > 0.4. BID and CTSC showed high binding strength (combined score = 0.908); strong interactions were also observed between BID–FAS (combined score = 0.873) and BID–FASLG (combined score = 0.867). Additionally, proteins such as GATA3, RORA, and CTSC had high connectivity in the network, indicating their key roles in candidate gene-related signaling pathways (detailed interactions in **Figure 8T**; **Supplementary Table 7**).

### 3.10 Single-cell analysis of non-immune cells in pancreatic cancer patients

Based on the GSE194247 dataset (focused on non-immune cell subsets of pancreatic cancer patients), we preprocessed and annotated cell subsets into six types (including Stellate) using marker genes from Seongryong Kim et al. (37) (**Supplementary Figures S7A–D****)**. Fibroblasts were further extracted, batch-corrected by Harmony, and re-clustered into seven subtypes using marker-based annotation (**Supplementary Figures S7E–H**).

**Figure 9:**
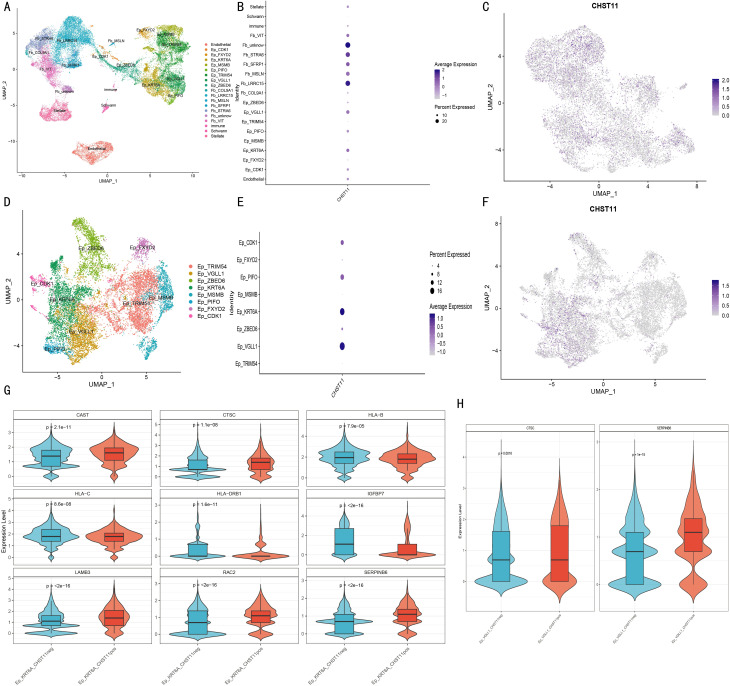
Single-cell analysis of non-immune cells in pancreatic cancer. **(A)** UMAP of 19 cellular subclusters; **(B)** Bubble plot of CHST11 expression across cell types; **(C)** UMAP of CHST11 distribution in fibroblasts; **(D)** UMAP of eight malignant epithelial subclusters; **(E, F)** Bubble and UMAP plots of CHST11 expression in malignant epithelial subclusters; **(G–H)** Violin plots of differential gene expression between CHST11^+^ and CHST11^-^ cells in Ep_KRT6A and Ep_VGLL1 subtypes.

Given Seongryong Kim et al.’s indication that malignant cells are mainly in epithelial cells, we focused on CHST11 expression in Epithelial cells. For annotation stability, Epithelial cells were re-corrected by Harmony and divided into 24 characteristic subsets. Dot plots were generated using the original marker genes; cell subset annotation and division were finalized by integrating dot plots and UMAP expression distributions of core genes (e.g., VGLL1, KRT6A) (**Supplementary Figure S8**), resulting in 19 cell subsets (**Figure 9A**).

Dot plots and UMAP showed CHST11 was mainly expressed in cancer-associated fibroblasts (**Figures 9B, C**) and in Ep_KRT6A/Ep_VGLL1 cells among malignant epithelial cells (**Figures 9D–F**). Differentially expressed genes (DEGs) between CHST11-positive and -negative cells of these two cell types were screened; Mendelian randomization (MR) was used to identify prognosis-related DEGs (**Supplementary Table 8**). MR-based screening of DEGs in Ep_VGLL1 and Ep_KRT6A cells confirmed higher CTSC expression in CHST11-positive cells of both malignant cell types (**Figures 9G, H**).

### 3.11 Spatial transcriptome analysis

**Figure 10:**
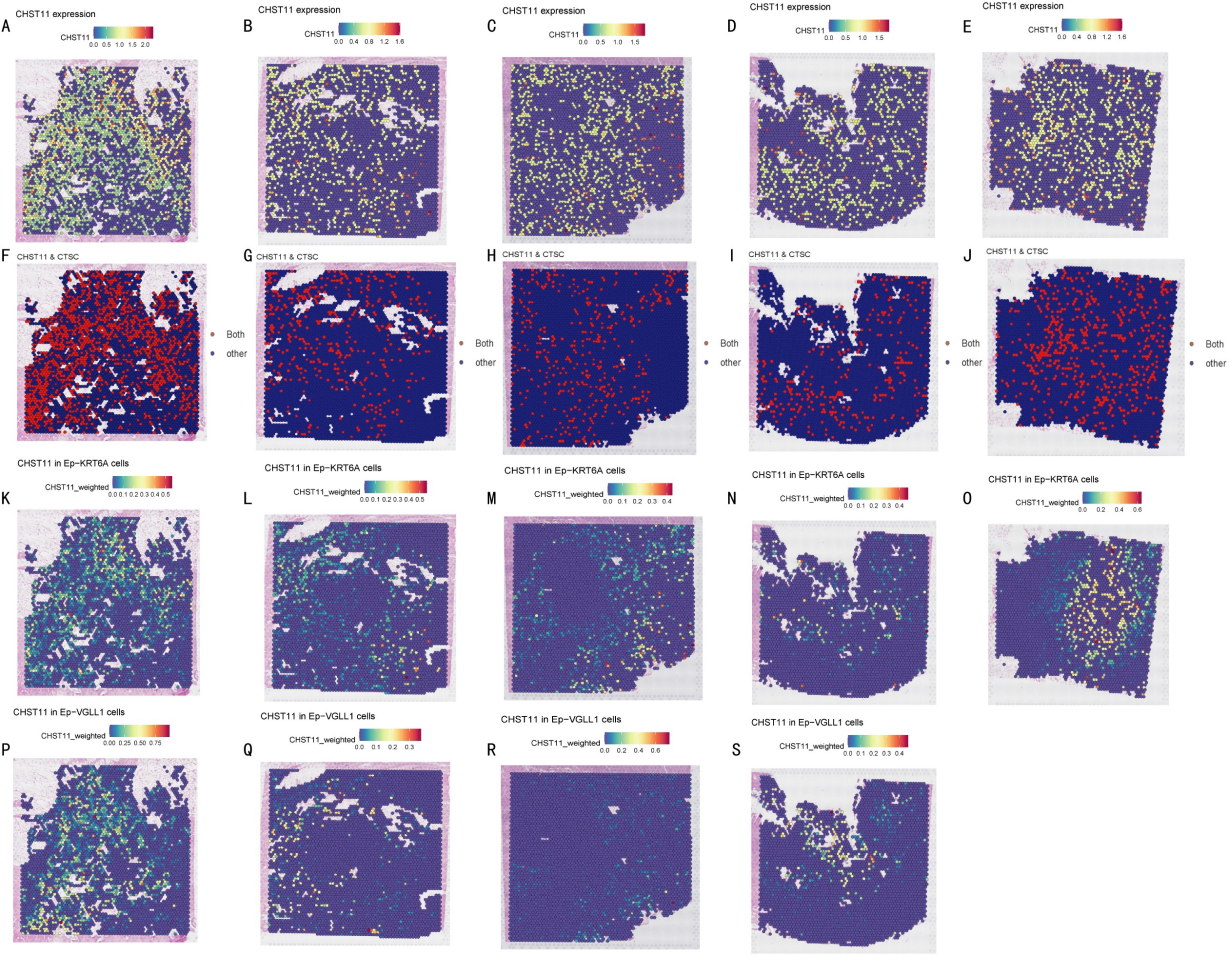
Spatial transcriptomics. **(A-E)** CHST11 expression across tumor sections from five pancreatic cancer patients (blue to red, 0–1.6); **(F-J)** Co-expression regions of CHST11 and CTSC (expression > 0.5) shown as red dots; **(K-O)** CHST11 expression in Ep-KRT6A cells; **(P-S)** CHST11 expression in Ep-VGLL1 cells.

Spatial transcriptomics (ST) analysis was conducted using matched data from five pancreatic cancer patients in the GSE194247 dataset. Results showed that CHST11 had high overall expression in pancreatic cancer tissues (**Figures 10A-E**) and shared significant co-expression regions with *CTSC* in spatial distribution (**Figures 10F-J**). Additionally, CHST11 exhibited obvious expression in both Ep_KRT6A (**Figures 10K-O**) and Ep_VGLL1 (**Figures 10P-S**), with significantly higher expression in Ep_KRT6A than in Ep_VGLL1.

### 3.12 Molecular docking

**Figure 11:**
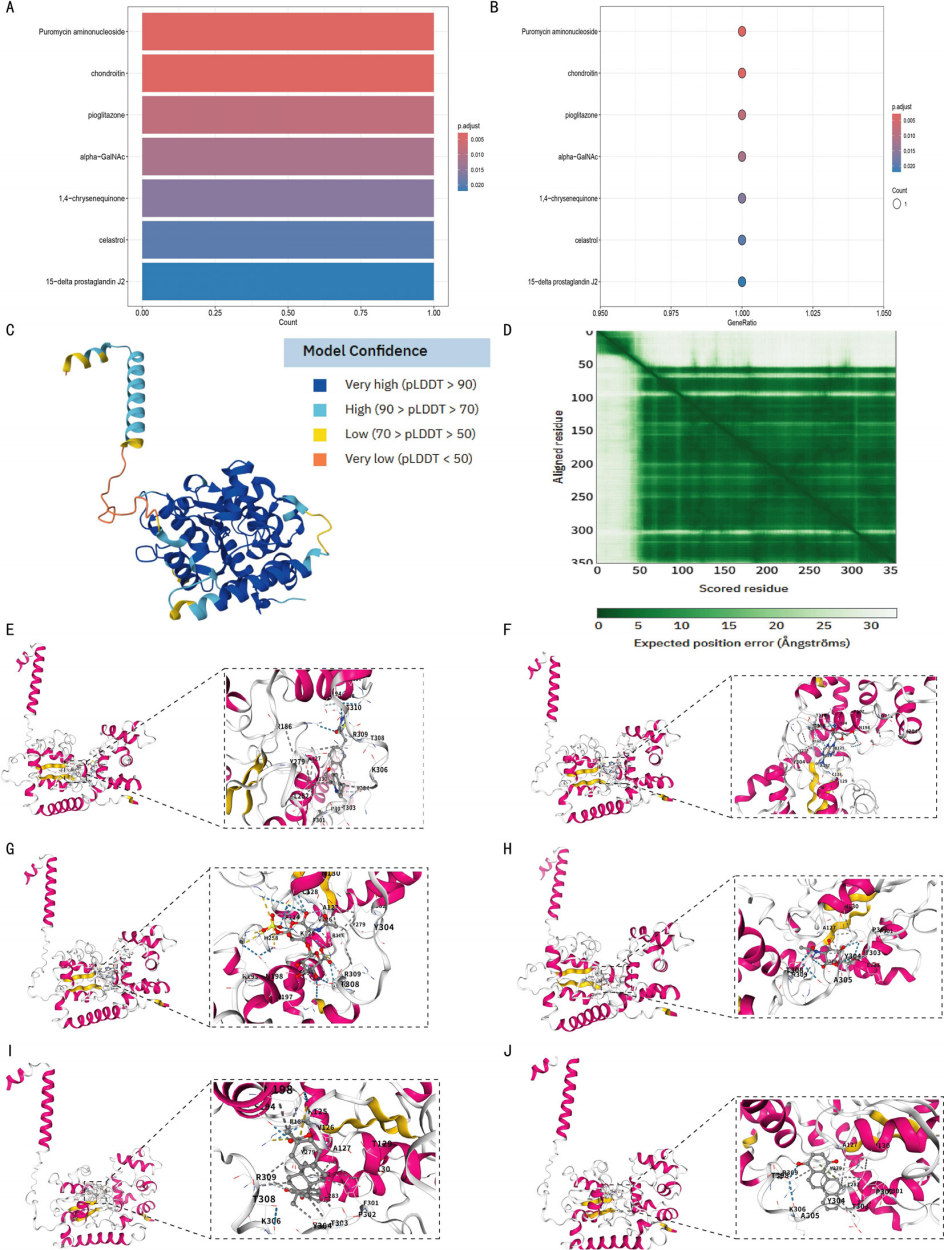
Structural prediction of CHST11 protein and molecular docking analysis of candidate drugs. **(A, B)** Enrichment results identifying seven candidate drugs predicted to interact with CHST11 (adjusted *p* < 0.05; bar/bubble plots); **(C)** Predicted 3D structure of CHST11 from AlphaFold, with confidence indicated by pLDDT scores; **(D)** Predicted alignment error (PAE) map showing positional uncertainty between residues; overall, CHST11 structure exhibited high confidence; **(E–J)** Molecular docking results showing binding modes of six candidate drugs with CHST11, with left panels showing overall binding sites and right panels showing magnified binding pockets.

Using p.adjust < 0.05 as the screening threshold, 7 drugs with significant interactions with the CHST11 gene were identified: Puromycin aminonucleoside, Chondroitin, Pioglitazone, Alpha-GalNAc, 1,4-Chrysenequinone, Celastrol, and 15-Delta prostaglandin J2 (**Figures 11A, B**). Additionally, AlphaFold-based structure prediction showed high overall confidence for the CHST11 protein (**Figures 11C, D**).

Molecular docking analysis was then performed to examine the binding of 6 of these drugs to the CHST11 protein. As shown in **Figures 11E-J**, the docking results included Pioglitazone (Vina score = –8.4), Puromycin aminonucleoside (Vina score = –7.5), Chondroitin (Vina score = –8.4), Alpha-GalNAc (Vina score = –6.0), Celastrol (Vina score = –8.8), and 1,4-Chrysenequinone (Vina score = –9.2).

### 3.13 qPCR and immunohistochemistry

**Figure 12:**
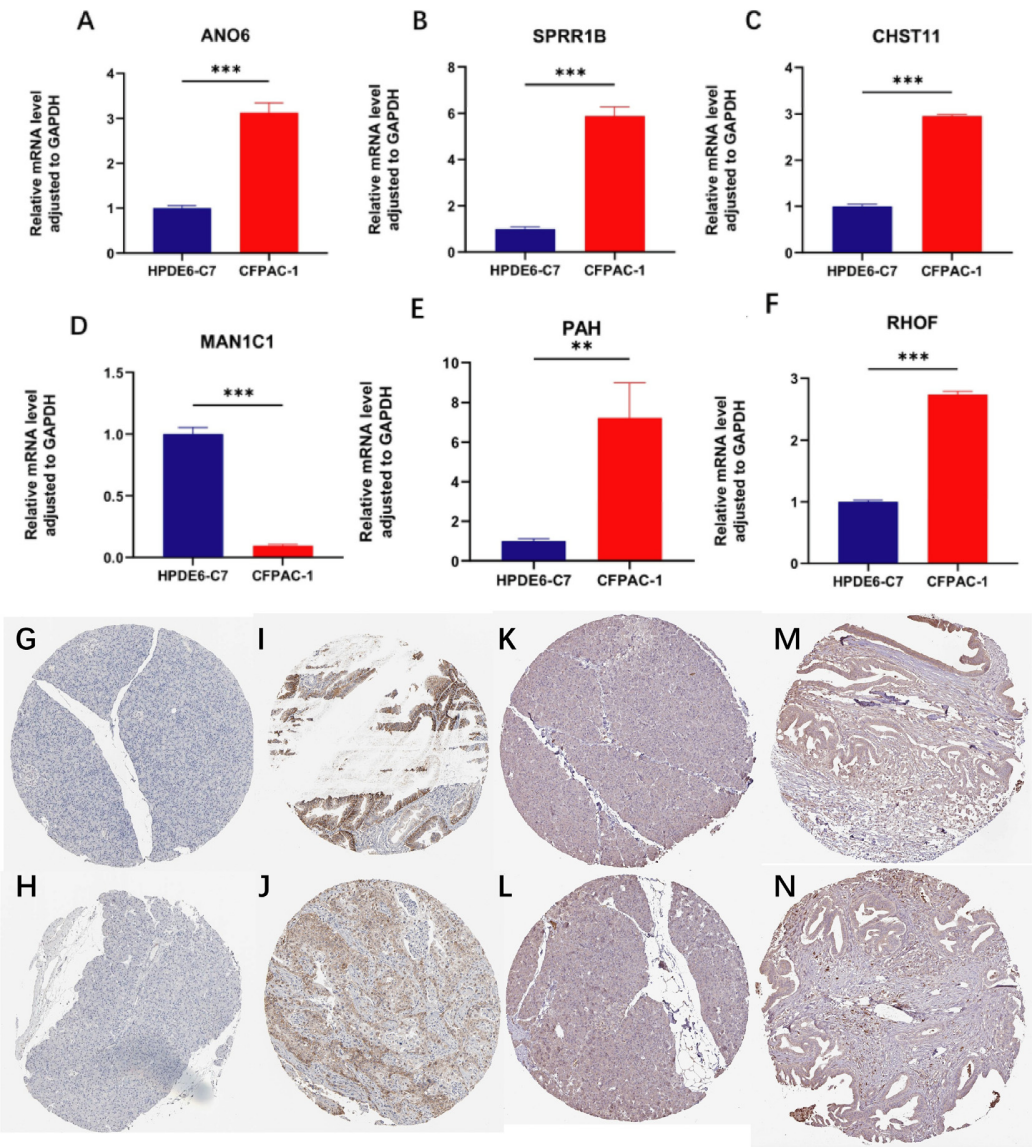
qPCR and immunohistochemistry analyses. **(A–F)** qPCR results showing differential expression of ANO6, SPRR1B, CHST11, PAH, RHOF, and MAN1C1 between normal pancreatic cell line HPDE6-C7 and pancreatic cancer cell line CFPAC-1 (**p < 0.01, ***p < 0.001); **(G–J)** Immunohistochemistry of SLC16A1 showing absent in in normal pancreatic tissue but moderate–high in pancreatic cancer; **(K–N)** Immunohistochemistry of CHST11 showing low expression in normal pancreas and moderate expression in pancreatic cancer tissues.

In this study, normal pancreatic cell line HPDE6-C7 and pancreatic cancer cell line CFPAC-1 were selected, and qPCR was performed on 7 model genes to compare their expression differences between normal pancreatic and pancreatic cancer tissues. Results showed significant differences in 6 genes: AN06, SPRR1B, CHST11, PAH and RHOF were significantly upregulated, while MAN1C1 was significantly downregulated (**Figures 12A-F**).

Further, immunohistochemical expression of these 7 genes was analyzed in the HPA database. SLC16A1 was undetectable in 2 normal pancreatic samples, but expressed in 11 out of 12 pancreatic cancer patients (6 with moderate-high expression, 1 with high expression) (**Figures 12G-J**). For CHST11, low expression was observed in 3 normal pancreatic tissues, while differential expression was found in pancreatic cancer tissues: of 8 patients, 2 were negative, 3 had weak expression, and 3 had moderate expression (**Figures 12K-N**).

## 4 Discussion

In previous bioinformatics studies, researchers often began with known gene sets associated with specific biological features and identified overlapping genes related to prognosis for further investigation—for example, the studies by Jinsong Liu et al. (43)and Peikai Ding et al. (44). In studies related to necroptosis in pancreatic cancer, researchers also typically adopted a similar approach. For instance, Longchen Yu et al. performed RNA sequencing on 5 pairs of pancreatic cancer and adjacent normal tissues to identify differentially expressed genes (DEGs), then integrated necroptosis-related genes to screen potential targets (45). Comparable approaches were adopted by Haichuan Liu et al. (46) and Hanna Belfrage et al. (47). In this study, we performed unsupervised clustering on 15 necroptosis-related genes in an integrated dataset to screen for potential key genes closely associated with necroptosis in pancreatic cancer.

Previous studies commonly used unsupervised clustering to classify molecular subgroups, screen DEGs, and further identify signature genes *via* prognostic analysis. For example, Cheng Zeng et al. divided patients into two LMF subgroups by unsupervised clustering, then used LASSO regression to screen prognosis-related genes from subgroup DEGs and explored their roles in patient prognosis (48). Similar strategies were applied by Cheng Zeng et al. (49)and ZhangPing Yu et al. (50). Although unsupervised clustering combined with differential analysis effectively identifies signature-related genes, single clustering often generates numerous DEGs. Previous studies typically used LASSO or univariate Cox regression to select a few prognostic signatures from hundreds of candidates for model construction. While this extracts core prognostic factors, it often ignores associations with clustering features and lacks analyses verifying biological relevance. In our study, we continuously traced the association between core factors (extracted stepwise *via* prognostic features) and the 15 necroptosis-related genes. Specifically, we first clustered samples into two NRGclusters based on the 15 genes and screened their DEGs. Next, 495 prognosis-related genes were selected from these DEGs *via* univariate Cox analysis, followed by a second clustering. Sankey diagram analysis evaluated signature similarity between subtypes from the two clusterings. Further LASSO regression identified 7 key prognostic genes, dividing patients into high- and low-risk groups. Results showed 11 of the 15 necroptosis-related genes had significant expression differences between risk groups; NRGclusterA highly overlapped with geneClusterB, both corresponding to the low-risk group. Focusing on CHST11 (the most significant prognostic gene), we found it correlated with most necroptosis-related genes, and necroptosis genes were highly expressed in CHST11-positive T cells. Unlike previous studies focusing solely on prognostic signatures, we verified associations with necroptosis genes after each screening and modeling step, ensuring biological consistency and mechanistic relevance of the selected signatures.

We found CHST11 correlated with poor prognosis in multiple pancreatic cancer prognostic datasets. Additionally, PCR, combined TCGA-GTEx analysis, and immunohistochemistry confirmed higher CHST11 expression in pancreatic cancer tissues and cells. Previous studies reported CHST11 overexpression in various cancers, typically correlating with poor prognosis. For example, high CHST11 protein expression is an independent poor prognostic factor in ovarian cancer (51); in clear cell renal cell carcinoma (ccRCC), recent studies linked high CHST11 to clinical stage, immune microenvironment features, and poor survival, and *in vitro* experiments showed it promotes tumor cell proliferation, migration, and invasion (52). Few studies have explored CHST11’s prognostic role and mechanisms in pancreatic cancer. Our study first performed immune infiltration analysis, finding CHST11 significantly correlated with macrophages, T cells, and dendritic cells. Consistent with this, CHST11 was highly expressed in T cells, NK cells, MDSCs, and cDC1s in mouse pancreatic cancer tissues. Furthermore, CHST11 expression was significantly higher in immunotherapy-treated groups than in untreated groups.

We found that in both naïve T cell_CHST11^+^ and T cell_CHST11^+^ populations, the average expression levels of necroptosis-related genes GATA3 and FAS were higher than those in CHST11^-^ cells, and the proportion of GATA3^+^ cells was significantly increased. Previous studies have demonstrated that GATA3 is a key transcription factor for Th2 cell differentiation, which inhibits Th1 differentiation by suppressing IL-12Rβ2 expression and maintains the Th2 phenotype through a positive feedback mechanism (53). These findings suggest that may participate in the regulation of naïve T-cell differentiation by modulating the expression of key genes such as GATA3. Additionally, T cell_CHST11^+^ populations showed significantly upregulated FASLG, GATA3, and TNFRSF1A, with a rising trend in FAS. Previous studies reported high-molecular-weight sulfated polysaccharides (e.g., heparin, heparan sulfate, dextran sulfate) enhance Fas-mediated T cell death (54). As CHST11 is a key enzyme for glycosaminoglycan sulfation, these results suggest CHST11^+^ naive T cells in pancreatic cancer may have "Th2 differentiation tendency" and promote T cell exhaustion by enhancing apoptosis sensitivity *via* the FAS/FASLG pathway.

We further explored CHST11’s association with non-immune cells in pancreatic cancer, finding it mainly localized to fibroblasts and epithelial cells. Numerous studies link CHST11 to fibroblasts: primary fibroblasts from Costello syndrome patients show reduced chondroitin-4-sulfate (C4S) and lower CHST11 mRNA/protein expression; oncogenic HRAS expression in normal fibroblasts inhibits CHST11, while interfering with oncogenic HRAS signaling in Costello syndrome fibroblasts upregulates CHST11 (55). Additionally, CHST11 promotes tumor malignancy and the production of fibrosis activators; TGF-β and INF-γ induce CHST11 expression in fibrosis models and regulate CHST11-related molecules, indicating CHST11-fibroblast associations in pulmonary fibrosis (56). In our study, we focused on pancreatic cancer malignant epithelial cells and found CHST11 was highly expressed in Ep_KRT6A and Ep_VGLL1 cells. High CTSC expression was observed in naive T cell_CHST11^+^, Ep_KRT6A_CHST11^+^, and Ep_VGLL1_CHST11^+^ cells. Mendelian randomization identified CTSC as an adverse factor for pancreatic cancer; TCGA data showed CTSC was significantly positively correlated with CHST11 and associated with poor prognosis. Previous studies reported increased CTSC expression in myeloid cells from normal pancreas to pancreatic squamous cell carcinoma (57) and its key role in islet carcinogenesis (58). Spatial transcriptomics analysis showed high CHST11 expression in Ep_KRT6A and significant co-localization between CHST11 and CTSC, suggesting they may synergistically promote pancreatic cancer progression.

In summary, one core innovation of this study is the establishment of a feature screening system combining "two consensus cluster analyses + continuous biological tracing." Verifying associations with necroptosis-related genes after each screening step alleviates the "signature dilution" issue common in previous studies that "directly combine survival analysis with single consensus clustering." Additionally, to our knowledge, this study is the first to systematically explore CHST11’s prognostic features in pancreatic cancer *via* single-cell omics, showing immunotherapy may upregulate CHST11 expression. It also preliminarily suggests CHST11 may regulate T cell differentiation and promote exhaustion. Meanwhile, CHST11 and CTSC may synergistically promote pancreatic cancer progression. Despite optimized design, this study has limitations: (1) External validation of CHST11’s prognostic features relied mainly on public databases, with potential selection bias due to pancreatic cancer heterogeneity and incomplete pathological subtype coverage; future studies should include subgroup and pathological subtype-specific analyses. (2) Despite batch effect correction, platform differences between TCGA and GEO and inherent technical noise in single-cell sequencing may introduce minor measurement errors. (3) External validation only focused on gene expression, lacking clinical variables and multi-center data, limiting external validity. (4) Although multi-omics provided clues for associations between CHST11 and GATA3/CTSC/FAS, conclusions rely mainly on bioinformatics correlation analyses; cellular and animal experiments are needed to validate core mechanisms and supplement causal evidence.

## 5 Conclusion

By means of multi-omics approaches, this study reveals that CHST11 is associated with necroptosis. Meanwhile, it identifies that this gene is linked to poor prognosis of pancreatic cancer, and this prognostic association is closely related to the role of CHST11 in T cells and malignant epithelial cells.

## Data Availability

The original contributions presented in the study are included in the article/**Supplementary Material**. Further inquiries can be directed to the corresponding author.

## Supplementary material

Supplementary Figure 1 Unsupervised clustering of necroptosis-related genes (NRGs). **(A–I)** Consensus matrix heatmaps for cluster numbers (k) = 1–9; **(J)** Cumulative distribution function (CDF) plot for k = 1–9; **(K)** Delta area plot of relative changes in CDF curve area; **(L)** Tracking plot of sample classification at different k values.

Supplementary Figure 2 Gene clustering based on prognosis-related differentially expressed genes (PRDEGs).**(A–I)** Consensus matrix heatmaps for k = 1–9; **(J)** CDF plot for k = 1–9; **(K)** Delta area plot of relative changes in CDF curve area; **(L)** Tracking plot of sample distribution at different k values.

Supplementary Figure 3 Tumor purity. No significant difference in tumor purity between high- and low-risk groups.

Supplementary Figure 4 TME-based PCA. Principal component analysis (PCA) based on tumor microenvironment (TME) features showed partial separation between high-risk (red circles) and low-risk (blue triangles) groups, with some overlap.

Supplementary Figure 5 Drug sensitivity. Drugs showing significantly different IC50 values between low- and high-risk groups.

Supplementary Figure 6 Preprocessing and clustering of single-cell data in pancreatic cancer tissues.**(A)** Data preprocessing; **(B)** PCA-based dimensionality reduction; **(C)** UMAP visualization of clusters; **(D)** UMAP distribution of the seven model genes.

Supplementary Figure 7 Single-cell analysis of non-immune cells.**(A–D)** Preprocessing, harmony reduction, UMAP visualization of 16 clusters, and bubble plots of marker genes; **(E–D)** Fibroblast reclustering and fibroblast marker gene expression across seven fibroblast subclusters.

Supplementary Figure 8 Single-cell analysis of epithelial cells.**(A–C)** Harmony-based reclustering, UMAP visualization of 25 epithelial subclusters, and bubble plots of marker genes; **(D)** Distribution of key marker genes such as VGLL1 across epithelial subclusters.

## References

[1] Cai J Chen H Lu M Zhang Y Lu B You L . Advances in the epidemiology of pancreatic cancer: Trends, risk factors, screening, and prognosis. Cancer letters. (2021) 520:1–11. doi: 10.1016/j.canlet.2021.06.027, PMID: 34216688

[2] Ribas A Wolchok JD . Cancer immunotherapy using checkpoint blockade. Sci (New York NY). (2018) 359:1350–5. doi: 10.1126/science.aar4060, PMID: 29567705 PMC7391259

[3] Gao W Wang X Zhou Y Wang X Yu Y . Autophagy, ferroptosis, pyroptosis, and necroptosis in tumor immunotherapy. Signal transduction targeted Ther. (2022) 7:196. doi: 10.1038/s41392-022-01046-3, PMID: 35725836 PMC9208265

[4] Wang H Zhou X Li C Yan S Feng C He J . The emerging role of pyroptosis in pediatric cancers: from mechanism to therapy. J Hematol Oncol. (2022) 15:140. doi: 10.1186/s13045-022-01365-6, PMID: 36209102 PMC9547461

[5] Holler N Zaru R Micheau O Thome M Attinger A Valitutti S . Fas triggers an alternative, caspase-8-independent cell death pathway using the kinase RIP as effector molecule. Nat Immunol. (2000) 1:489–95. doi: 10.1038/82732, PMID: 11101870

[6] He S Wang L Miao L Wang T Du F Zhao L . Receptor interacting protein kinase-3 determines cellular necrotic response to TNF-alpha. Cell. (2009) 137:1100–11. doi: 10.1016/j.cell.2009.05.021, PMID: 19524512

[7] Zhang DW Shao J Lin J Zhang N Lu BJ Lin SC . RIP3, an energy metabolism regulator that switches TNF-induced cell death from apoptosis to necrosis. Sci (New York NY). (2009) 325:332–6. doi: 10.1126/science.1172308, PMID: 19498109

[8] Sun L Wang H Wang Z He S Chen S Liao D . Mixed lineage kinase domain-like protein mediates necrosis signaling downstream of RIP3 kinase. Cell. (2012) 148:213–27. doi: 10.1016/j.cell.2011.11.031, PMID: 22265413

[9] Zhao J Jitkaew S Cai Z Choksi S Li Q Luo J . Mixed lineage kinase domain-like is a key receptor interacting protein 3 downstream component of TNF-induced necrosis. Proc Natl Acad Sci United States America. (2012) 109:5322–7. doi: 10.1073/pnas.1200012109, PMID: 22421439 PMC3325682

[10] Zhao Z Liu H Zhou X Fang D Ou X Ye J . Necroptosis-related lncRNAs: predicting prognosis and the distinction between the cold and hot tumors in gastric cancer. J Oncol. (2021) 2021:6718443. doi: 10.1155/2021/6718443, PMID: 34790235 PMC8592775

[11] Zhong B Wang Y Liao Y Liang J Wang K Zhou D . MLKL and other necroptosis-related genes promote the tumor immune cell infiltration, guiding for the administration of immunotherapy in bladder urothelial carcinoma. Apoptosis: an Int J programmed Cell death. (2023) 28:892–911. doi: 10.1007/s10495-023-01830-8, PMID: 37000317 PMC10232593

[12] Liu D Xu S Chang T Ma S Wang K Sun G . Predicting prognosis and distinguishing cold and hot tumors in bladder urothelial carcinoma based on necroptosis-associated lncRNAs. Front Immunol. (2022) 13:916800. doi: 10.3389/fimmu.2022.916800, PMID: 35860239 PMC9289196

[13] Tao Y Feng F Luo X Reihsmann CV Hopkirk AL Cartailler JP . CNTools: A computational toolbox for cellular neighborhood analysis from multiplexed images. PloS Comput Biol. (2024) 20:e1012344. doi: 10.1371/journal.pcbi.1012344, PMID: 39196899 PMC11355562

[14] Ito K Murphy D . Application of ggplot2 to pharmacometric graphics. CPT: pharmacometrics Syst Pharmacol. (2013) 2:e79. doi: 10.1038/psp.2013.56, PMID: 24132163 PMC3817376

[15] Johnson WE Li C Rabinovic A . Adjusting batch effects in microarray expression data using empirical Bayes methods. Biostatistics (Oxford England). (2007) 8:118–27. doi: 10.1093/biostatistics/kxj037, PMID: 16632515

[16] Wilkerson MD Hayes DN . ConsensusClusterPlus: a class discovery tool with confidence assessments and item tracking. Bioinf (Oxford England). (2010) 26:1572–3. doi: 10.1093/bioinformatics/btq170, PMID: 20427518 PMC2881355

[17] Love MI Huber W Anders S . Moderated estimation of fold change and dispersion for RNA-seq data with DESeq2. Genome Biol. (2014) 15:550. doi: 10.1186/s13059-014-0550-8, PMID: 25516281 PMC4302049

[18] Hänzelmann S Castelo R Guinney J . GSVA: gene set variation analysis for microarray and RNA-seq data. BMC Bioinf. (2013) 14:7. doi: 10.1186/1471-2105-14-7, PMID: 23323831 PMC3618321

[19] Barbie DA Tamayo P Boehm JS Kim SY Moody SE Dunn IF . Systematic RNA interference reveals that oncogenic KRAS-driven cancers require TBK1. Nature. (2009) 462:108–12. doi: 10.1038/nature08460, PMID: 19847166 PMC2783335

[20] Aleksander SA Balhoff J Carbon S Cherry JM Drabkin HJ Ebert D . The gene ontology knowledgebase in 2023. Genetics. (2023) 224:iyad031. doi: 10.1093/genetics/iyad031, PMID: 36866529 PMC10158837

[21] Kanehisa M Goto S . KEGG: kyoto encyclopedia of genes and genomes. Nucleic Acids Res. (2000) 28:27–30. doi: 10.1093/nar/28.1.27, PMID: 10592173 PMC102409

[22] Brunson JC . ggalluvial: layered grammar for alluvial plots. J Open Source software. (2020) 5:2017. doi: 10.21105/joss.02017, PMID: 36919162 PMC10010671

[23] Posta M Győrffy B . Analysis of a large cohort of pancreatic cancer transcriptomic profiles to reveal the strongest prognostic factors. Clin Trans science. (2023) 16:1479–91. doi: 10.1111/cts.13563, PMID: 37260110 PMC10432876

[24] Bartha Á Győrffy B . TNMplot.com: A web tool for the comparison of gene expression in normal, tumor and metastatic tissues. Int J Mol Sci. (2021) 22:2622. doi: 10.3390/ijms22052622, PMID: 33807717 PMC7961455

[25] Newman AM Liu CL Green MR Gentles AJ Feng W Xu Y . Robust enumeration of cell subsets from tissue expression profiles. Nat Methods. (2015) 12:453–7. doi: 10.1038/nmeth.3337, PMID: 25822800 PMC4739640

[26] Yoshihara K Shahmoradgoli M Martínez E Vegesna R Kim H Torres-Garcia W . Inferring tumour purity and stromal and immune cell admixture from expression data. Nat Commun. (2013) 4:2612. doi: 10.1038/ncomms3612, PMID: 24113773 PMC3826632

[27] Mayakonda A Lin DC Assenov Y Plass C Koeffler HP . Maftools: efficient and comprehensive analysis of somatic variants in cancer. Genome Res. (2018) 28:1747–56. doi: 10.1101/gr.239244.118, PMID: 30341162 PMC6211645

[28] Geeleher P Cox N Huang RS . pRRophetic: an R package for prediction of clinical chemotherapeutic response from tumor gene expression levels. PloS One. (2014) 9:e107468. doi: 10.1371/journal.pone.0107468, PMID: 25229481 PMC4167990

[29] Hao Y Hao S Andersen-Nissen E Mauck WM 3rd Zheng S Butler A . Integrated analysis of multimodal single-cell data. Cell. (2021) 184:3573–87.e29. doi: 10.1016/j.cell.2021.04.048, PMID: 34062119 PMC8238499

[30] Jin S Guerrero-Juarez CF Zhang L Chang I Ramos R Kuan CH . Inference and analysis of cell-cell communication using CellChat. Nat Commun. (2021) 12:1088. doi: 10.1038/s41467-021-21246-9, PMID: 33597522 PMC7889871

[31] Qiu X Mao Q Tang Y Wang L Chawla R Pliner HA . Reversed graph embedding resolves complex single-cell trajectories. Nat Methods. (2017) 14:979–82. doi: 10.1038/nmeth.4402, PMID: 28825705 PMC5764547

[32] Hemani G Zheng J Elsworth B Wade KH Haberland V Baird D . The MR-Base platform supports systematic causal inference across the human phenome. eLife. (2018) 7:e34408. doi: 10.7554/eLife.34408, PMID: 29846171 PMC5976434

[33] Tang Z Kang B Li C Chen T Zhang Z . GEPIA2: an enhanced web server for large-scale expression profiling and interactive analysis. Nucleic Acids Res. (2019) 47:W556–w60. doi: 10.1093/nar/gkz430, PMID: 31114875 PMC6602440

[34] Szklarczyk D Kirsch R Koutrouli M Nastou K Mehryary F Hachilif R . The STRING database in 2023: protein-protein association networks and functional enrichment analyses for any sequenced genome of interest. Nucleic Acids Res. (2023) 51:D638–d46. doi: 10.1093/nar/gkac1000, PMID: 36370105 PMC9825434

[35] Shannon P Markiel A Ozier O Baliga NS Wang JT Ramage D . Cytoscape: a software environment for integrated models of biomolecular interaction networks. Genome Res. (2003) 13:2498–504. doi: 10.1101/gr.1239303, PMID: 14597658 PMC403769

[36] Li JH Liu S Zhou H Qu LH Yang JH . starBase v2.0: decoding miRNA-ceRNA, miRNA-ncRNA and protein-RNA interaction networks from large-scale CLIP-Seq data. Nucleic Acids Res. (2014) 42:D92–7. doi: 10.1093/nar/gkt1248, PMID: 24297251 PMC3964941

[37] Kim S Leem G Choi J Koh Y Lee S Nam SH . Integrative analysis of spatial and single-cell transcriptome data from human pancreatic cancer reveals an intermediate cancer cell population associated with poor prognosis. Genome Med. (2024) 16:20. doi: 10.1186/s13073-024-01287-7, PMID: 38297291 PMC10832111

[38] Yoo M Shin J Kim J Ryall KA Lee K Lee S . DSigDB: drug signatures database for gene set analysis. Bioinf (Oxford England). (2015) 31:3069–71. doi: 10.1093/bioinformatics/btv313, PMID: 25990557 PMC4668778

[39] Wu T Hu E Xu S Chen M Guo P Dai Z . clusterProfiler 4.0: A universal enrichment tool for interpreting omics data. Innovation (Cambridge (Mass)). (2021) 2:100141. doi: 10.1016/j.xinn.2021.100141, PMID: 34557778 PMC8454663

[40] Kim S Chen J Cheng T Gindulyte A He J He S . PubChem in 2021: new data content and improved web interfaces. Nucleic Acids Res. (2021) 49:D1388–d95. doi: 10.1093/nar/gkaa971, PMID: 33151290 PMC7778930

[41] Varadi M Bertoni D Magana P Paramval U Pidruchna I Radhakrishnan M . AlphaFold Protein Structure Database in 2024: providing structure coverage for over 214 million protein sequences. Nucleic Acids Res. (2024) 52:D368–d75. doi: 10.1093/nar/gkad1011, PMID: 37933859 PMC10767828

[42] Liu Y Yang X Gan J Chen S Xiao ZX Cao Y . CB-Dock2: improved protein-ligand blind docking by integrating cavity detection, docking and homologous template fitting. Nucleic Acids Res. (2022) 50:W159–w64. doi: 10.1093/nar/gkac394, PMID: 35609983 PMC9252749

[43] Liu J Lu Y Dai Y Shen Y Zeng C Liu X . A comprehensive analysis and validation of cuproptosis-associated genes across cancers: Overall survival, the tumor microenvironment, stemness scores, and drug sensitivity. Front Genet. (2022) 13:939956. doi: 10.3389/fgene.2022.939956, PMID: 36105090 PMC9465292

[44] Ding P Pei S Qu Z Yang Y Liu Q Kong X . Single-cell sequencing unveils mitophagy-related prognostic model for triple-negative breast cancer. Front Immunol. (2024) 15:1489444. doi: 10.3389/fimmu.2024.1489444, PMID: 39559367 PMC11570810

[45] Yu L Guo Q Li Y Mao M Liu Z Li T . CHMP4C promotes pancreatic cancer progression by inhibiting necroptosis via the RIPK1/RIPK3/MLKL pathway. J advanced Res. (2025) 77:653–668. doi: 10.1016/j.jare.2025.01.040, PMID: 39870301 PMC12627866

[46] Liu H Li Z Zhang L Zhang M Liu S Wang J . Necroptosis-related prognostic model for pancreatic carcinoma reveals its invasion and metastasis potential through hybrid EMT and immune escape. Biomedicines. (2023) 11:1738. doi: 10.3390/biomedicines11061738, PMID: 37371833 PMC10296367

[47] Belfrage H Kuuliala K Kuuliala A Mustonen H Puolakkainen P Kylänpää L . Circulating necroptosis markers in chronic pancreatitis and pancreatic cancer: Associations with diagnosis and prognostic factors. Pancreatology: Off J Int Assoc Pancreatology (IAP) [et al]. (2024) 24:1229–36. doi: 10.1016/j.pan.2024.11.016, PMID: 39613683

[48] Zeng C Wang J Zhao S Wei Y Qi Y Liu S . Multi-cohort validation of a lipid metabolism and ferroptosis-associated index for prognosis and immunotherapy response prediction in hormone receptor-positive breast cancer. Int J Biol Sci. (2025) 21:3968–92. doi: 10.7150/ijbs.113213, PMID: 40612672 PMC12223777

[49] Zeng C Xu C Liu S Wang Y Wei Y Qi Y . Integrated bulk and single-cell transcriptomic analysis unveiled a novel cuproptosis-related lipid metabolism gene molecular pattern and a risk index for predicting prognosis and antitumor drug sensitivity in breast cancer. Discover Oncol. (2025) 16:318. doi: 10.1007/s12672-025-02044-x, PMID: 40085377 PMC11909392

[50] Ding C Yu Z Zhu J Li X Dai M He. Q . Construction and validation of a necroptosis-related gene signature for predicting prognosis and tumor microenvironment of pancreatic cancer. Dis markers. (2022) 2022:9737587. doi: 10.1155/2022/9737587, PMID: 35756487 PMC9214653

[51] Oliveira-Ferrer L Heßling A Trillsch F Mahner S Milde-Langosch K . Prognostic impact of chondroitin-4-sulfotransferase CHST11 in ovarian cancer. Tumour biology: J Int Soc Oncodevelopmental Biol Med. (2015) 36:9023–30. doi: 10.1007/s13277-015-3652-3, PMID: 26084610

[52] Hu W Chen Y Zhang L Guo X Wei X Shao Y . Effect of CHST11, a novel biomarker, on the biological functionalities of clear cell renal cell carcinoma. Sci Rep. (2024) 14:7704. doi: 10.1038/s41598-024-58280-8, PMID: 38565604 PMC10987617

[53] Tamauchi H Terashima M Ito M Maruyama H Ikewaki N Inoue M . Evidence of GATA-3-dependent Th2 commitment during the *in vivo* immune response. Int Immunol. (2004) 16:179–87. doi: 10.1093/intimm/dxh026, PMID: 14688073

[54] Manero F Ljubic-Thibal V Moulin M Goutagny N Yvin JC Arrigo AP . Stimulation of Fas agonistic antibody-mediated apoptosis by heparin-like agents suppresses Hsp27 but not Bcl-2 protective activity. Cell Stress chaperones. (2004) 9:150–66. doi: 10.1379/csc-16r.1, PMID: 15497502 PMC1065295

[55] Klüppel M Samavarchi-Tehrani P Liu K Wrana JL Hinek A . C4ST-1/CHST11-controlled chondroitin sulfation interferes with oncogenic HRAS signaling in Costello syndrome. Eur J Hum genetics: EJHG. (2012) 20:870–7. doi: 10.1038/ejhg.2012.12, PMID: 22317973 PMC3400726

[56] Li CH Chan MH Chang YC Hsiao M . The CHST11 gene is linked to lung cancer and pulmonary fibrosis. J Gene Med. (2022) 24:e3451. doi: 10.1002/jgm.3451, PMID: 36181245

[57] Zhao X Li H Lyu S Zhai J Ji Z Zhang Z . Single-cell transcriptomics reveals heterogeneous progression and EGFR activation in pancreatic adenosquamous carcinoma. Int J Biol Sci. (2021) 17:2590–605. doi: 10.7150/ijbs.58886, PMID: 34326696 PMC8315026

[58] Gocheva V Zeng W Ke D Klimstra D Reinheckel T Peters C . Distinct roles for cysteine cathepsin genes in multistage tumorigenesis. Genes Dev. (2006) 20:543–56. doi: 10.1101/gad.1407406, PMID: 16481467 PMC1410800

