# Supplementary figures and images for: Prognostic differential subpopulation classification and immunotherapy response prediction in pancreatic cancer patients based on the gene features of necrotizing apoptosis
Source: Front Immunol. 2025 Nov 19;16:1592231. doi: 10.3389/fimmu.2025.1592231 (PMC12672444; doi:10.3389/fimmu.2025.1592231)

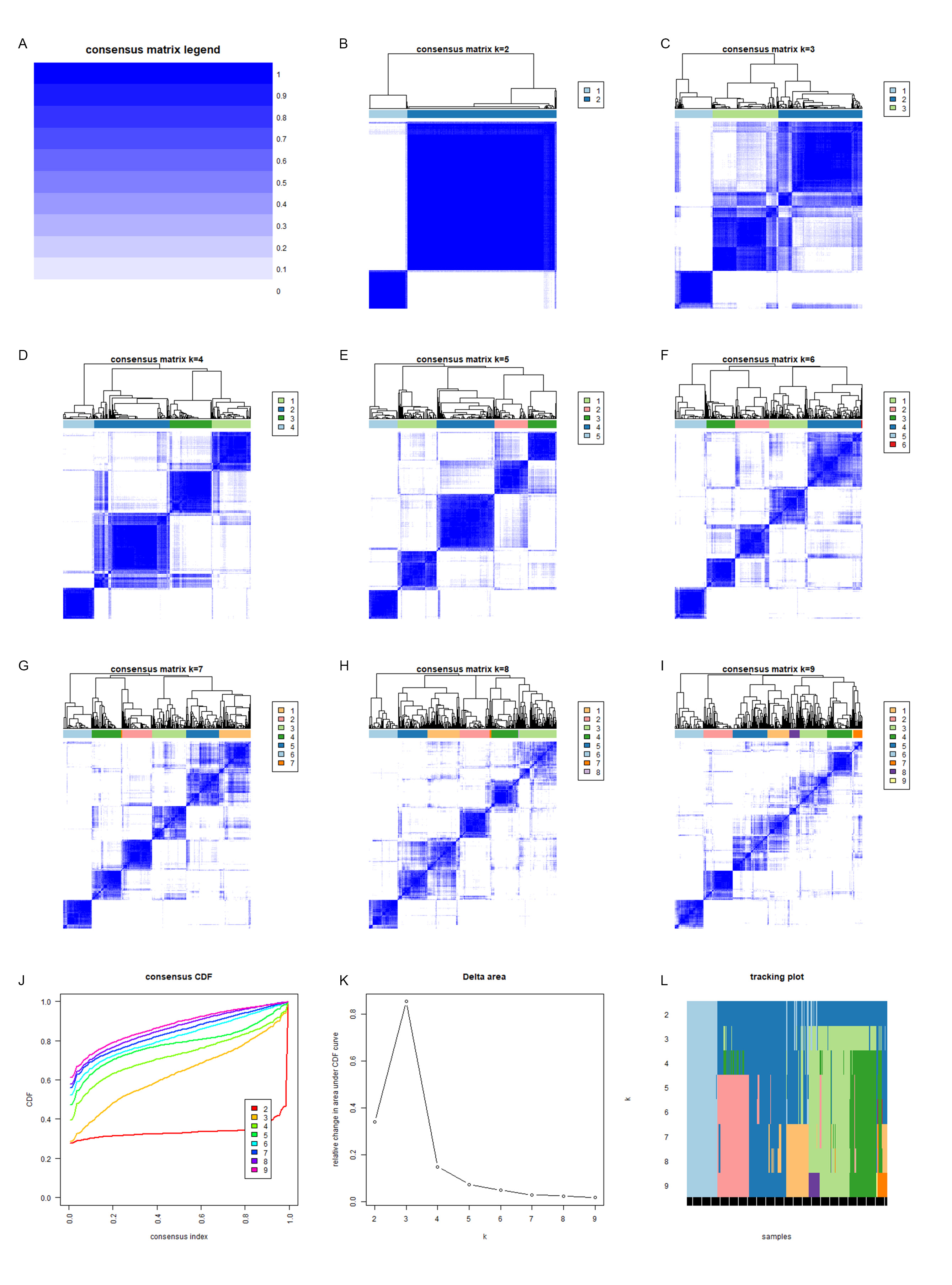

Supplement: Supplementary Figure 1 — Unsupervised clustering of necroptosis-related genes (NRGs). (A–I) Consensus matrix heatmaps for cluster numbers (k) = 1–9; (J) Cumulative distribution function (CDF) plot for k = 1–9; (K) Delta area plot of relative changes in CDF curve area; (L) Tracking plot of sample classification at different k values. [file Image1.jpeg]

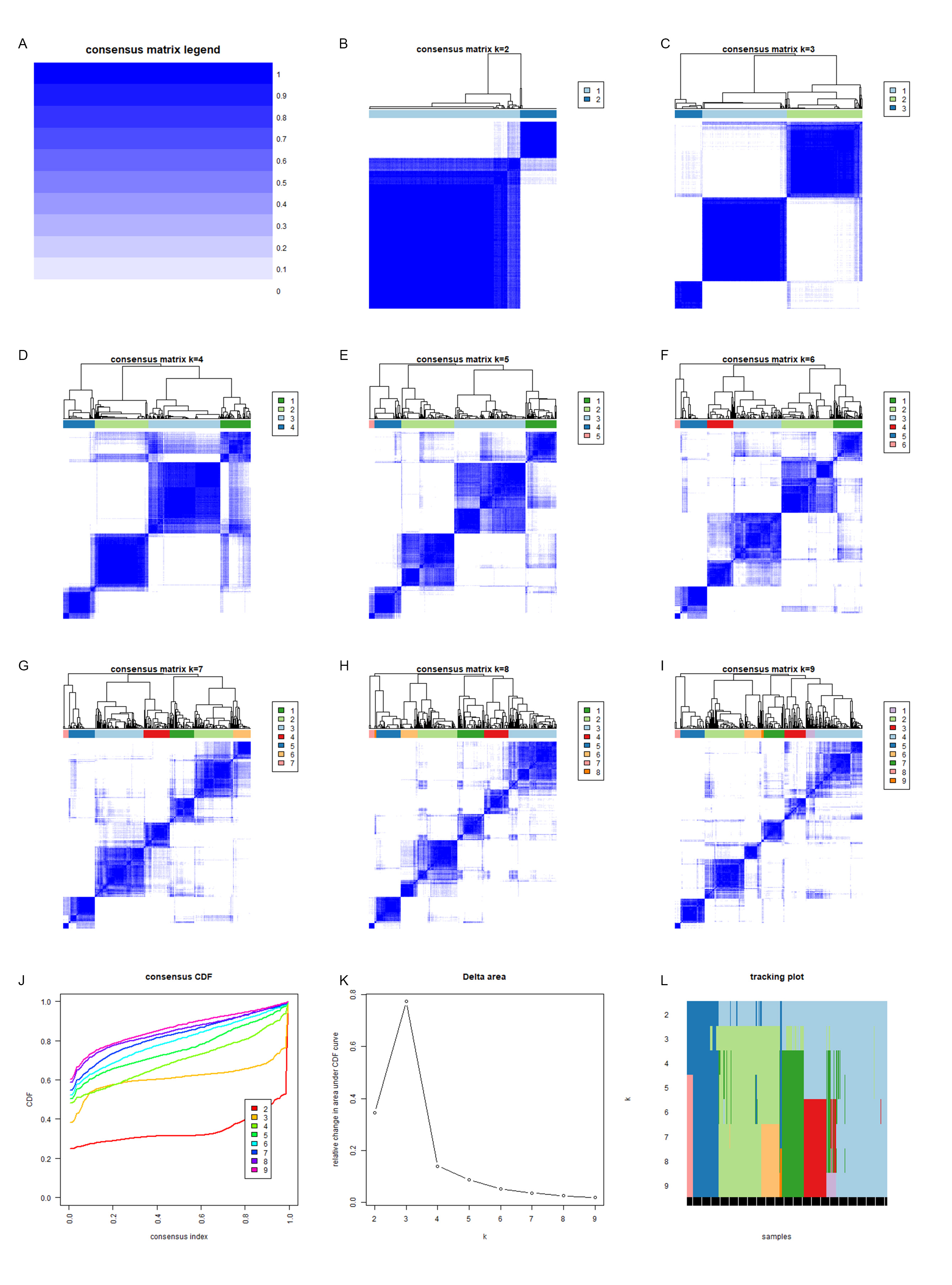

Supplement: Supplementary Figure 2 — Gene clustering based on prognosis-related differentially expressed genes (PRDEGs).(A–I) Consensus matrix heatmaps for k = 1–9; (J) CDF plot for k = 1–9; (K) Delta area plot of relative changes in CDF curve area; (L) Tracking plot of sample distribution at different k values. [file Image2.jpeg]

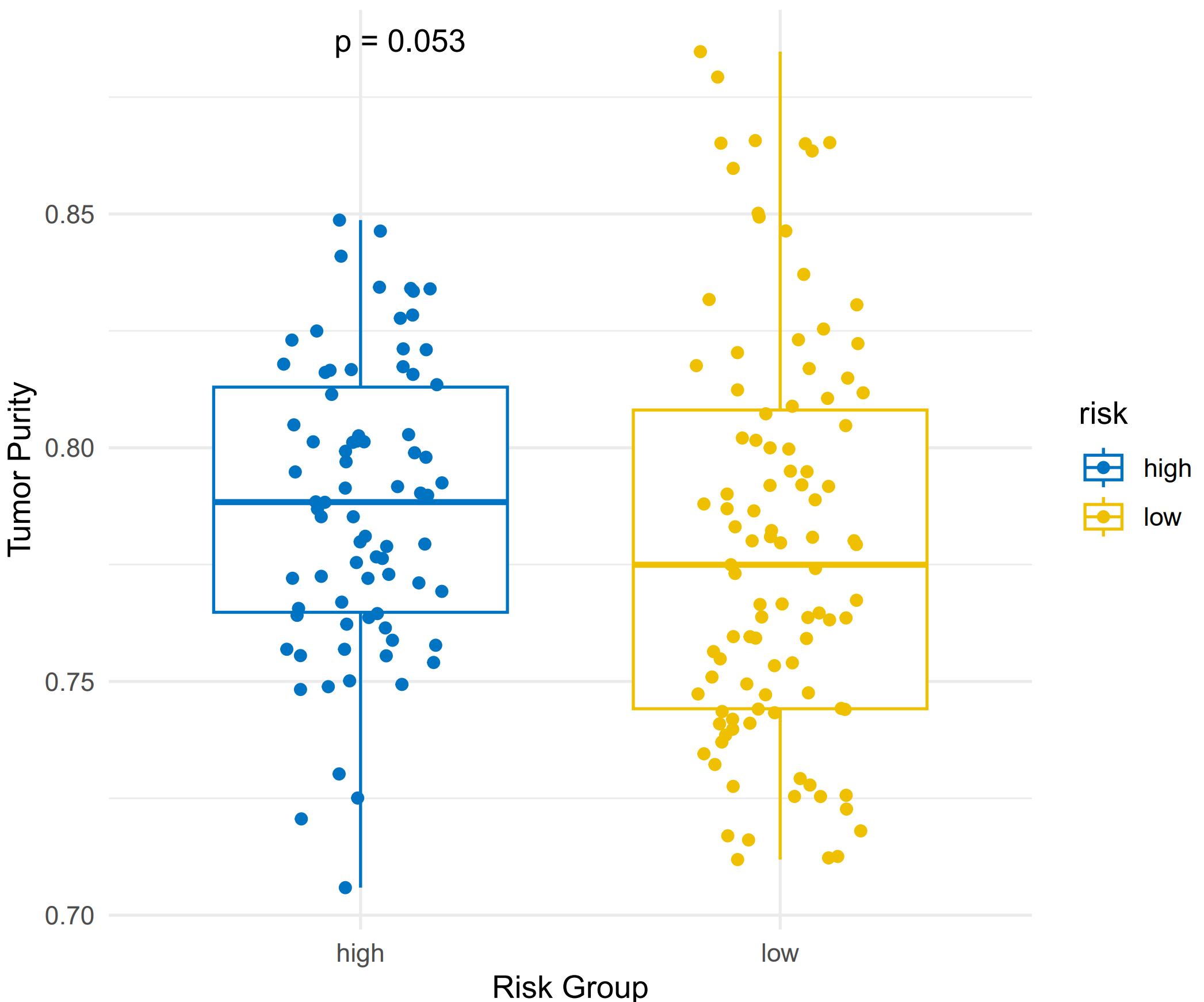

Supplement: Supplementary Figure 3 — Tumor purity. No significant difference in tumor purity between high- and low-risk groups. [file Image3.png]

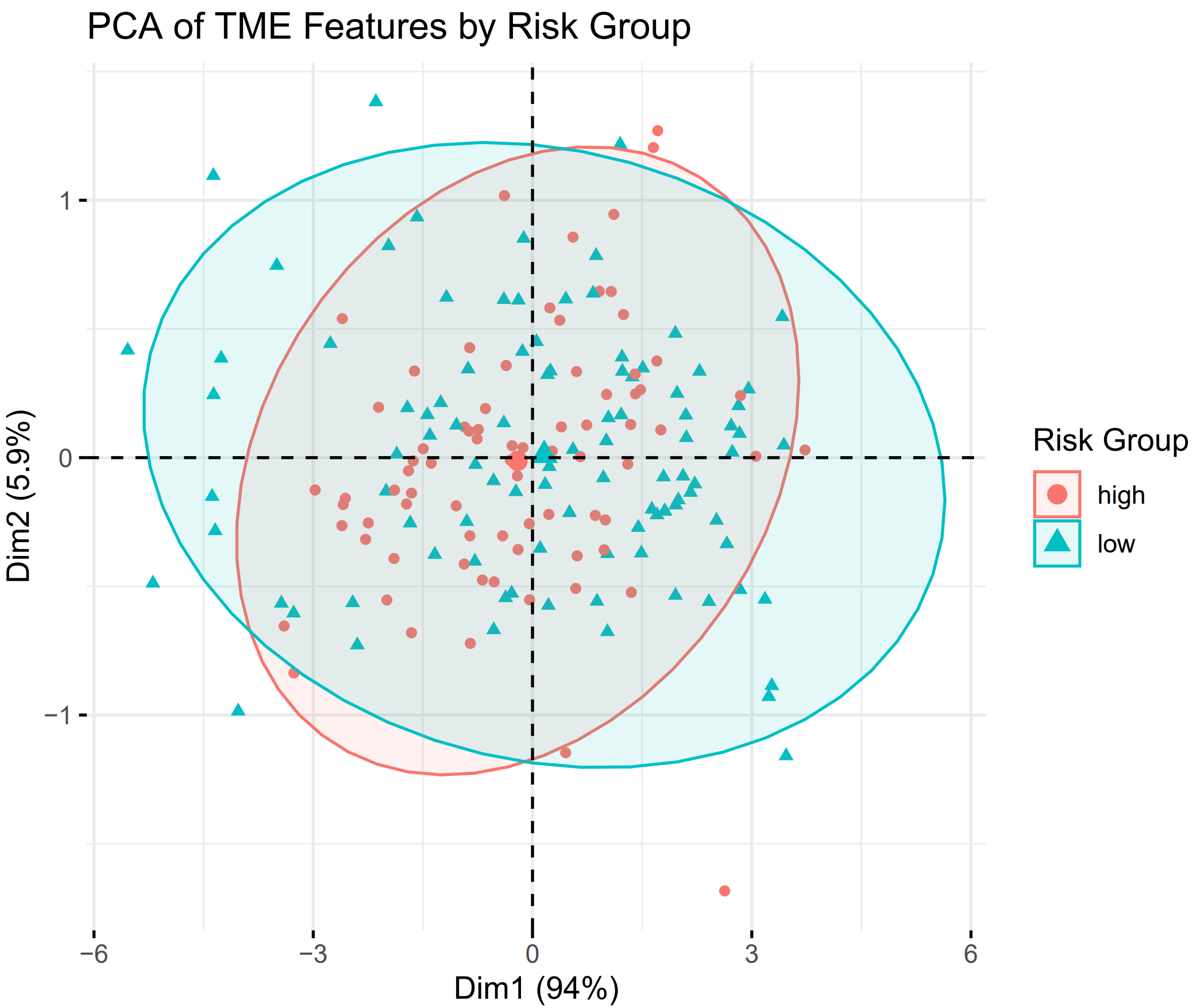

Supplement: Supplementary Figure 4 — TME-based PCA. Principal component analysis (PCA) based on tumor microenvironment (TME) features showed partial separation between high-risk (red circles) and low-risk (blue triangles) groups, with some overlap. [file Image4.png]

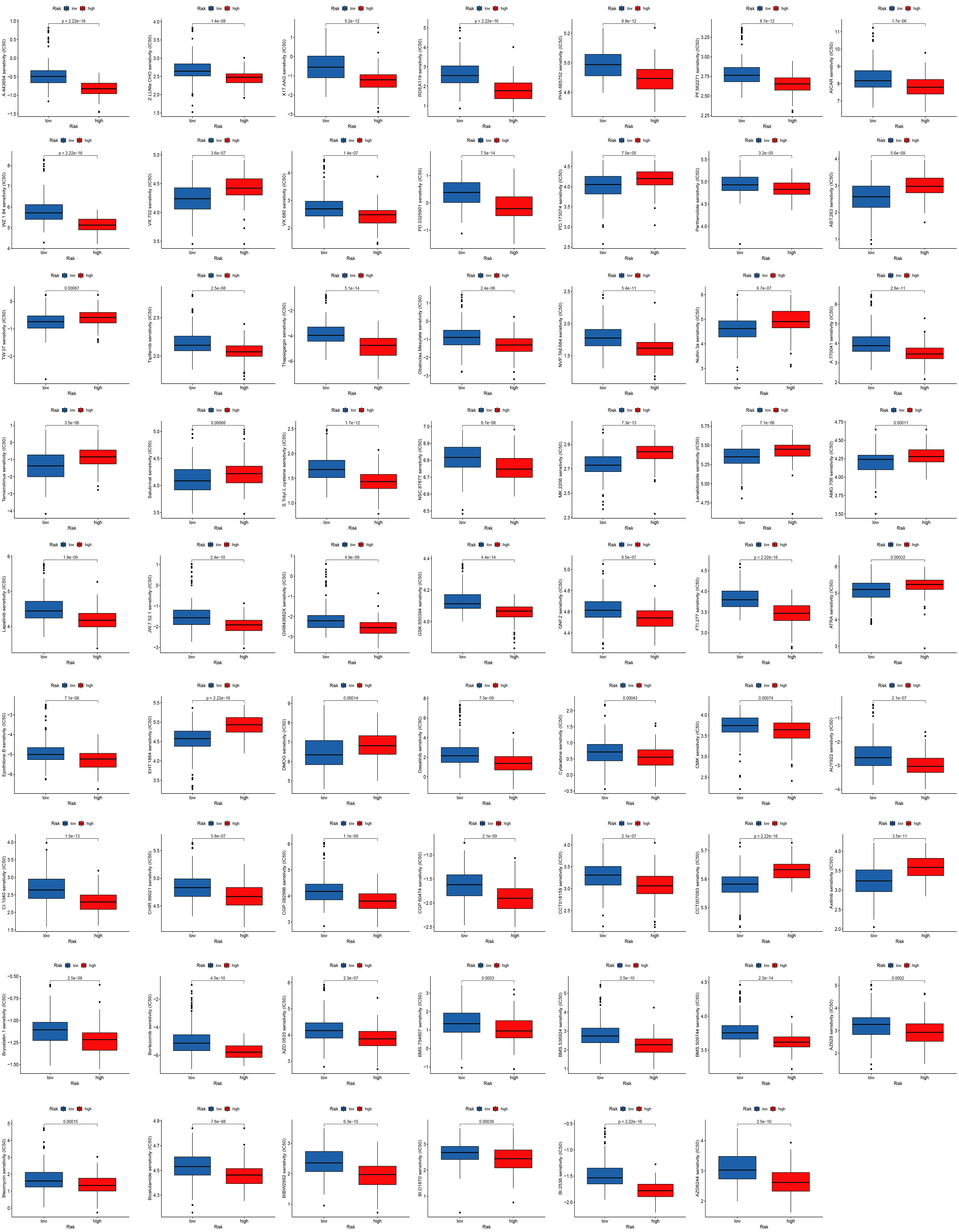

Supplement: Supplementary Figure 5 — Drug sensitivity. Drugs showing significantly different IC50 values between low- and high-risk groups. [file Image5.png]

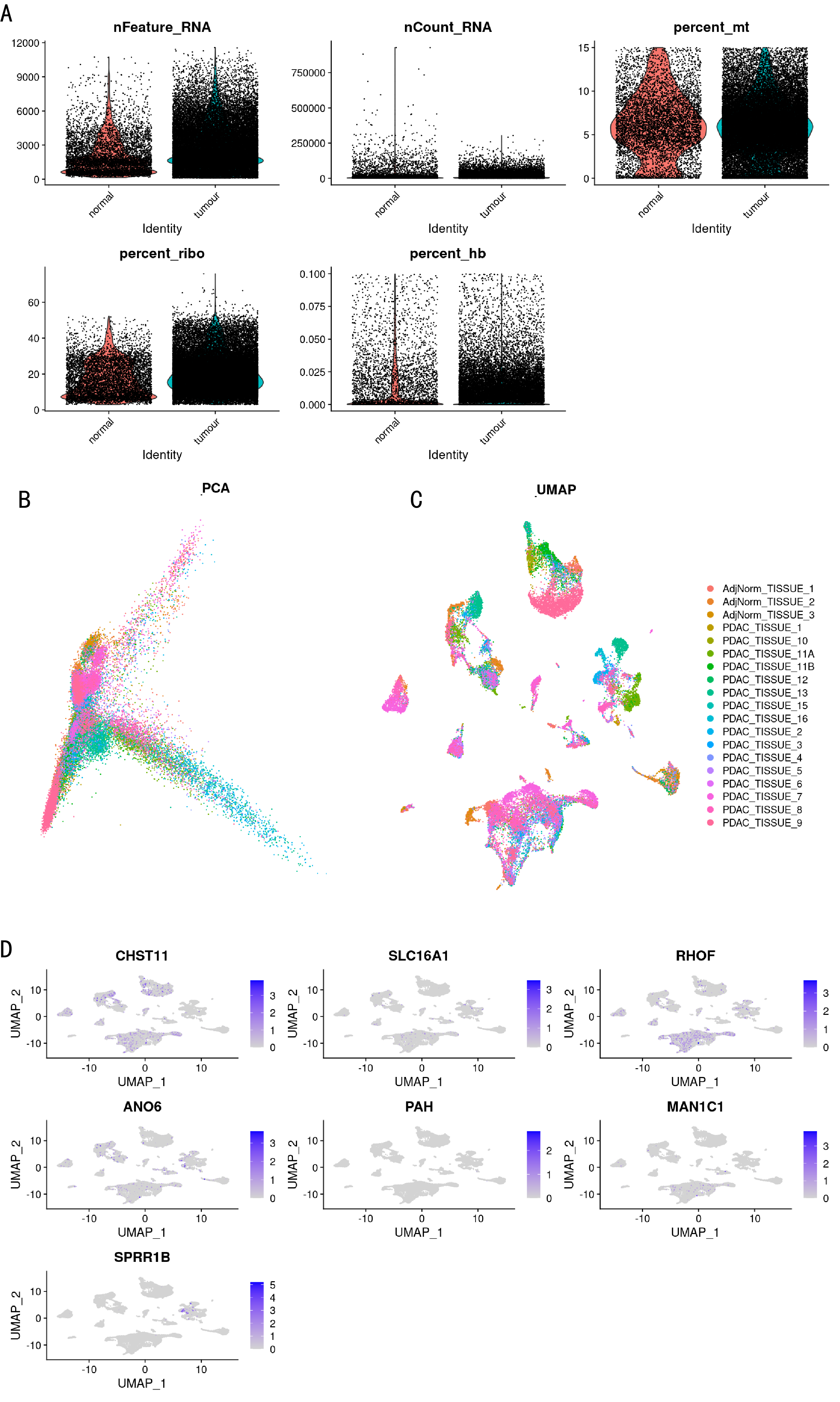

Supplement: Supplementary Figure 6 — Preprocessing and clustering of single-cell data in pancreatic cancer tissues.(A) Data preprocessing; (B) PCA-based dimensionality reduction; (C) UMAP visualization of clusters; (D) UMAP distribution of the seven model genes. [file Image6.png]

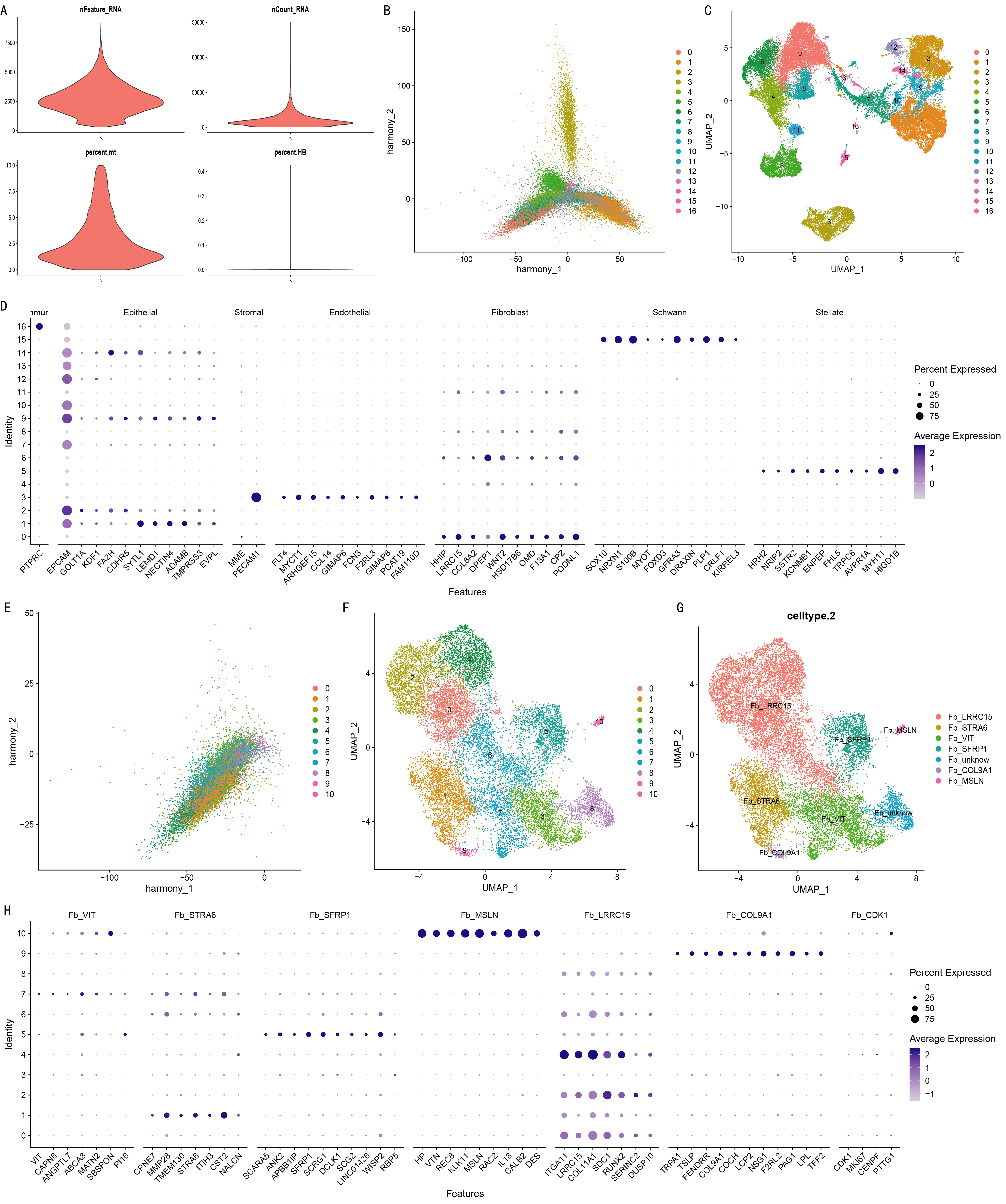

Supplement: Supplementary Figure 7 — Single-cell analysis of non-immune cells.(A–D) Preprocessing, harmony reduction, UMAP visualization of 16 clusters, and bubble plots of marker genes; (E–D) Fibroblast reclustering and fibroblast marker gene expression across seven fibroblast subclusters. [file Image7.png]

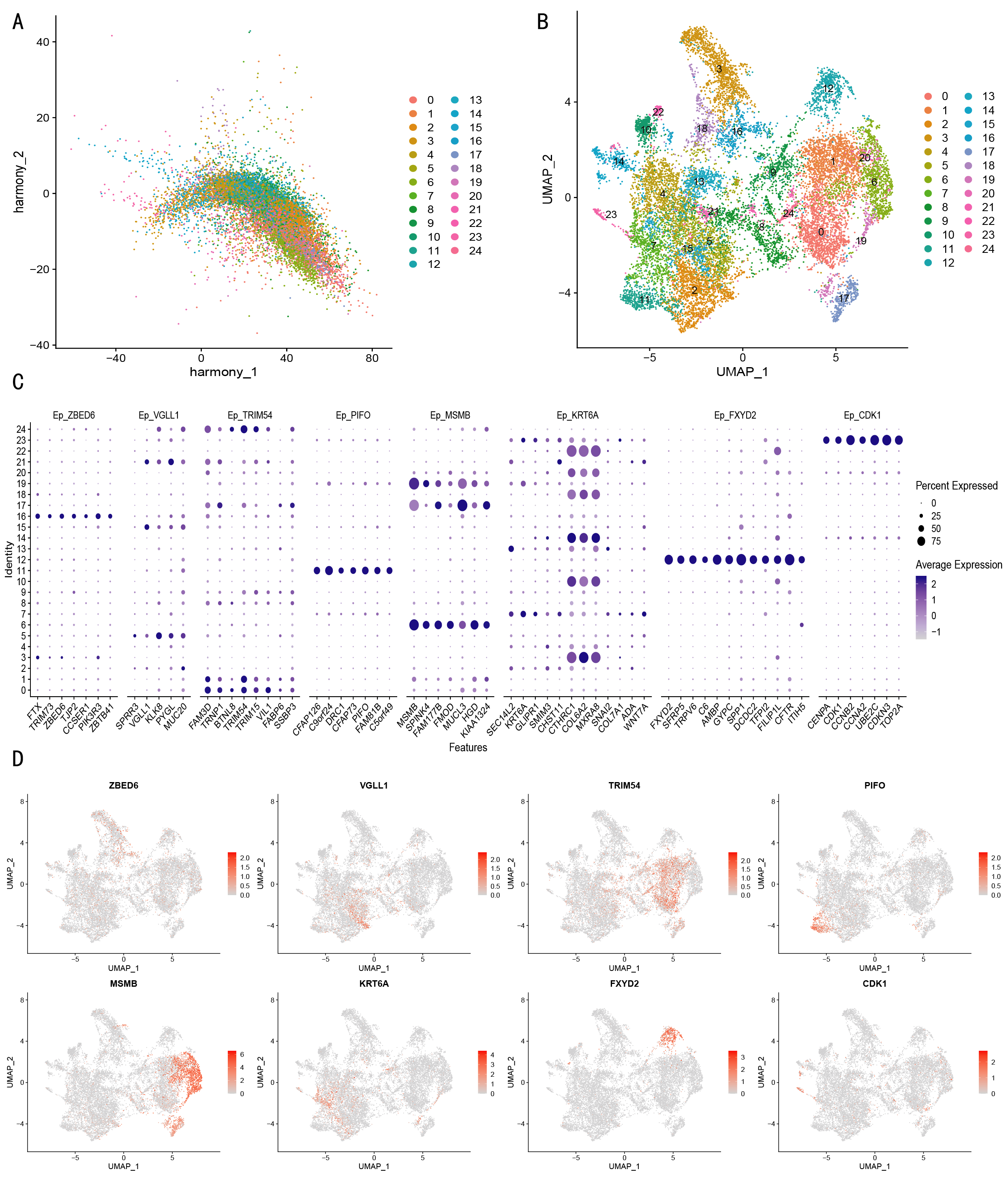

Supplement: Supplementary Figure 8 — Single-cell analysis of epithelial cells.(A–C) Harmony-based reclustering, UMAP visualization of 25 epithelial subclusters, and bubble plots of marker genes; (D) Distribution of key marker genes such as VGLL1 across epithelial subclusters. [file Image8.png]
